# Supplementary material for: Redefining species concepts for the Pennsylvanian scissor tooth shark, Edestus
Source: PLoS One. 2019 Sep 4;14(9):e0220958. doi: 10.1371/journal.pone.0220958 (PMC6726245; doi:10.1371/journal.pone.0220958)
Supplement: Attachment — Submitted filename: PONE-D-19-17544_JK.pdf [file pone.0220958.s004.pdf]

Manuscript Click here to download Manuscript Edestus - final.docx

Redefining species concepts for the Pennsylvanian scissor tooth shark, *Edestus*

Leif Tapanila[1,2*], and Jesse Pruitt[3]

Email: tapaleif@isu.edu

[1] Department of Geosciences, 921 S. 8th Ave., Idaho State University, Pocatello, ID 83209-8072, USA

[2] Earth Science Division, 921 S. 8th Ave., Idaho Museum of Natural History, Pocatello, ID 83209-8096, USA

[3] Idaho Virtualization Lab, 921 S. 8th Ave., Idaho Museum of Natural History, Pocatello, ID 83209-8096, USA

*Corresponding author

E-mail: tapaleif@isu.edu (LT)

Abstract

This study reevaluates the tooth morphology used to define species within the genus *Edestus* (Chondrichthyes, Euchondrocephali). Known as the scissor tooth shark, *Edestus* produced a unique dentition of spiraled tooth families positioned in the symphysis of the upper and lower jaws. Morphometric analysis of more than 200 ejected teeth and intact spiral tooth whorls demonstrates that teeth from the upper and lower whorls differ in shape and ontogeny. Comparison of these data to the type specimens of 13 existing species reduces the number of morphologically distinct *Edestus* to just four species and refines the stratigraphic occurrence and expansion of the group.

E. triserratus has a narrow bullet-shaped crown that points anteriorly and has roots of intermediate length. *E. minor* crowns have a wider triangular base, whereas the crowns of *E. heinrichi* form nearly equant triangles and are supported by an elongated root. *E. vorax* which also has roughly equant triangular crowns, has short and deep roots and is only known from very large specimens that are distinct from the growth series of *E. heinrichi*.

Edestus first appears in England during the latest Bashkirian, a few million years after its most closely resembling genus *Lestrodus*. Diversification and range expansion of *Edestus* coincides with the Moscovian transgression that flooded Laurentia and the Russian platform.

Introduction

Chondrichthyans belonging to the Edestoidea evolved some of the most unusual dental arrangements among vertebrates [1, 2]. The most conspicuous teeth are located in the symphysis of the lower jaw, forming a whorl of sharp, cutting crowns, best exemplified by *Helicoprion* Karpinsky 1899 [3, 4]. In *Edestus*, both the upper and lower jaws supported symphyseal whorls. Hay [5] first described a specimen showing the articulated anterior part of *Edestus* having two differently shaped, curved whorls of teeth. Nearly a century later, Zangerl and Jeremiah [6] documented at least three more specimens indicating dimorphism between the upper and lower whorls, but they stopped short of quantifying those differences or placing them in context of the cranium. CT scanning and 2D xrays of Zangerl and Jeremiah's specimens confirm a dimorphic upper and lower whorl and reveal their function as opposing cutting surfaces in the mandibular arch [2]. Although much has been gleaned about the form and function of *Edestus* from these specimens, the systematics of *Edestus* remain complicated by the fact that nearly all specimens of *Edestus* whorls lack cranial context or are recovered as individual teeth disaggregated from the whorl.

An *Edestus* whorl is composed of multiple teeth with long V-shaped roots that are stacked *en echelon* like shingles (Fig 1). Teeth are generated at the posterior and grow to the anterior where they are ejected out of the mouth. Teeth are preserved either as single ejected elements, or they are stacked *en echelon* together as part of a whorl containing up to a dozen crowns. According to Zangerl [1] and supported by Tapanila et al. [2], lower whorls generally have greater curvature than upper whorls, and they both form a spiral that, each, could have produced ~40 teeth during the lifetime of an animal.

Fig 1. Morphology and terminology of *Edestus* whorl and tooth.

(A) Merisitc measures and (B) geometric landmarks (LM) for individual teeth. (C) Multiple teeth comprise a whorl and are ejected from the anterior end as illustrated. W = crown width; H = crown height; uh = upper height; uw = upper width; D = distance from crown point to point.

As is the case for many Paleozoic chondrichthyans, cranial and post-cranial fossils are uncommon, therefore all 13 species of *Edestus* [7, 8] are defined solely by characters of the tooth crown. Moreover, nearly all species are defined by one specimen, and frequently by one partial crown lacking root material. Taxonomies based on single and incomplete dentitions are vulnerable to narrowly defined species that cannot account for the ontogenetic or environmental variations that might affect dental morphology within a species. Such was found to be the case in the edestoid genus *Helicoprion*, where morphometric analysis identified taxobases that resulted in the synonymization of most species founded on single or incomplete specimens [9]. *Edestus* is that much more challenging, considering that *Edestus* teeth are the product of two tooth-families, one from the upper whorl and one from the lower. Once ejected, it is difficult to know if a solitary tooth originated from an upper or lower whorl, and there is no reason to assume both upper and lower teeth have identical shape properties.

Formal revision of *Edestus* taxonomy is well overdue. Although previous reviews on *Edestus* have suggested revised synonymies, they invariably are based on qualitative assessment of few specimens (e.g., [10, 11, 6, 12, 8]). A common

observation from most authors is that species concepts for *Edestus* are too narrowly defined, and that most fit within two informally recognized end-members. The "symmetric" group has more equant triangular crowns oriented nearly perpendicular to the blade, whereas the "asymmetric" group has narrow crowns that slant anteriorly. Itano [8] demonstrated that the holotype of *E. minor* had been overlooked for nearly a century and may necessitate revision of several *Edestus* taxa, however, a perceived lack of specimens has thus far hampered any formal attempt (e.g., [7]). Here we aim to rectify the longstanding systematics dilemma by reporting on two extensive collections that provide hundreds of teeth to capture the ontogenetic series and define species concepts for both symmetric and asymmetric *Edestus* groups. This new dataset of measurements is compared against holotype and referred specimens of established *Edestus* species, and includes specimens known to have associated upper and lower whorls (Table 1). Taxonomic revision provides a clearer history on the range expansion and jaw morphologies of the unusual scissor tooth shark.

Table 1. Type specimens and figure references for previously defined *Edestus* species considered in this study.

Crown shape	Revised species	specimen number	Types and Previous designation	Image reference
Symmetric	*E. heinrichi*	USNM V 182450	Holotype *E. crenulatus*	This study, Fig 7K
	E. heinrichi	AMNH 488 cast	Cast of holotype *E. heinrichi*	This study, Fig 7J
	E. heinrichi	PIN RAN 1988/2	Holotype *E. karpinskyi*	Lebedev, 2001 [13], Pl. 44, fig. 7b
	E. heinrichi	n/a	Holotype *E. protopirata*	Trautschold 1879 [14], Pl. VI, fig. 8a
	E. heinrichi	USNM 6049	Holotype *E. serratus*	This study, Fig 7L
	E. vorax	AMNH 225	Holotype	This study, Fig 7O

			E. giganteus	
	E. vorax	ANSP 9899	Holotype<br>*E. vorax*	Leidy, 1856 [15]
Asymmetric	*E. minor*	AMNH FF477	Holotype, broken<br>*E. minor*	Itano, 2014 [8], figure 10
	E. minor	USNM 7255	Holotype<br>*E. mirus*	This study, Fig 7F
	E. minor	GSM 49368	Type<br>*E. pringlei*	This study, Fig 7C
	E. triserratus	GSM 31410	Holotype<br>*E. triserratus*	This study, Fig 7E
	E. triserratus	PIN RAN 2804/726	Holotype<br>*E. kolomnensis*	Lebedev, 2001 [13], Pl. 44, fig. 5
	E. triserratus	TSNiGR 11/1865	Holotype<br>*E. minusculus*	Karpinsky 1899 [3], Pl. IV, fig. 13

Material and Methods

The two primary collections of *Edestus* used in this study were assembled by Zangerl (FMNH) and Jeremiah (ANSP). The Zangerl collection includes dozens of specimens recovered from shale quarries of the Staunton and Linton formations in Illinois. Zangerl and Richardson [16] detail the excavation and description of the Mecca and Lloyd quarries, wherein hundreds of fish fossil were recovered from shallow marine transgressive deposits of Desmoinesian age. Most specimens are single teeth, ~~and~~ include several intact whorls. Specimens embedded in shale matrix were imaged with xray digital photography. Results generated from xray and photographic images yield overlapping measurements, validating the use of these two methods. Rare examples of associated cranial material (e.g., [6]) were examined to identify upper and lower whorl morphology in context of the jaw. Both asymmetric and symmetric teeth are present in the collection.

The Jeremiah collection includes half a dozen whorls and more than 40 specimens of complete or broken individual teeth. Although the specimens were collected without geological description, we have unpublished correspondence from Zangerl (pers. comm., 2003) indicating they were recovered from collapsed shale rocks that formed the roofs of abandoned coal mines. Presumably, these shales record marine or estuarine flooding deposits above coal marshes, similar to the fossils from the Zangerl collection. All specimens are from the Carbondale Formation in Illinois and are Desmoinesian in age. Only symmetric-crowned teeth comprise the Jeremiah collection.

In addition to these two collections, type and referred specimens of *Edestus* species were analyzed, giving our measured dataset a total of 140 specimens that include 211 measured tooth crowns. Updated stratigraphic and geographic summaries of *Edestus* add to previous summaries [17, 12], and use regional correlations to present occurrences in the global Pennsylvanian time scale [18].

Morphological analysis of *Edestus* followed meristic and geometric approaches. Linear dimensions and ratios summarized in Fig 1 were measured from photographs, xrays, or directly from specimens. Taxobases found to be helpful in describing the edestoid, *Helicoprion* [9] were assessed for their utility in defining *Edestus* species. Curvature of the whorl was assessed by measuring the angle between crowns (e.g., insertion angle: [9]) and distance between crown tips (D). The angle between two crowns is estimated by drawing lines that bisect each crown, and measuring the acute angle between them. Plotting cumulative distance and angle gives an estimated radius of curvature for the occlusal curve of the whorl. Sixteen whorls with intact series of crowns are presented, including holotypes for *E. mirus*, *E. crenulatus*, *E. serratus*, and

E. heinrichi. The holotype of *E. mirus* and the specimen FMNH PF8047 are unique in providing both upper and lower whorls in association.

Geometric morphometry was used to examine crown shape in 98 specimens of *Edestus*. Using tpsDig v. 2.16, three type 2 landmarks (LM1, LM20, LM39) were identified on the crown and these were connected with two series of equidistant semilandmarks to approximate the shape of the cutting surface (39 points total). Crown shapes were rotated and scaled using a full Procrustes superposition prior to generating covariance matrices and principle components analysis in MorphoJ v.1.05c. Results of the PCA identify shape parameters that are most significant in distinguishing specimens and when plotted according to crown size may identify ontogenetic series within the population of specimens in the analysis. All type specimens are included in the geometric analysis except the holotypes of *E. vorax* (ANSP 9899) and *E. triserratus* (GSM31410), which lack the crown apex, landmark 20. These specimens are included later in the methodology following meristic approximation to the geometric ordination. Recommendation for synonymy follows International Code of Zoological Nomenclature Article 23 giving priority to senior synonyms [19].

Institutional abbreviations for specimens as follows:

ACM, Beneski Museum of Natural History, Amherst College, Amherst, U.S.A.; **AMNH**, American Museum of Natural History, New York, U.S.A.; **ANSP**, Academy of Natural Sciences Museum, Philadelphia, U.S.A.; **CAS**, Chicago Academy of Science, Chicago, U.S.A.; **DMNH**, Denver Museum of Nature and Science, Denver, U.S.A.; **FMNH**, Field Museum of Natural History, Chicago, U.S.A.; **GSM**, British Geological Survey, Keyworth, Great Britain; **ISM**, Illinois State Museum, Springfield, U.S.A.; **KGS**, Kentucky

Geological Survey, Lexington, U.S.A.; **PIN**, Palaeontological Institute of the Russian Academy of Sciences, Moscow, Russia; **TMM**, Vertebrate Paleontology Laboratory, University of Texas, Austin, U.S.A.; **TSNiGR**, Central Research Geological Museum, St.-Petersburg, Russia; **USNM**, National Museum of Natural History, U.S.A.

Results

Morphometry and Establishment of Taxobases

Description and measures of the whorl

Edestus whorls all share several traits. The cutting (occlusal) surface forms a convex series of serrated crowns. Crown size decreases anteriorly, but in many specimens teeth within a single whorl are nearly the same size. Tooth roots are incrementally longer from posterior to anterior, until the last two or three teeth, which all have equal root length. The base of the whorl either has an outline that is shallowly or deeply concave (Fig 1). The base is interrupted by a subtle convexity (bulge) located halfway along the whorl; its position is further anterior in whorls with teeth having shorter roots. Anterior to the bulge is a scar marking the attachment point of the most recently ejected tooth. In associated specimens, the lower whorl displays greater curvature, especially in the concave base of the whorl, whereas the upper whorl has a comparatively straighter base. Whorls with symmetric teeth include the largest specimens of *Edestus* (e.g., holotype of *E. giganteus,* AMNH 225), and include concave and straight-based whorls. Straight-based whorls are less common in the asymmetric-

toothed group; only two specimens are known in this study (Jillson and CAS), whereas all others have broadly concave whorls.

Geometry of the whorl is quantified in two ways, the insertion angle and occlusal curvature (Fig 2). Insertion angle varies greatly among pairs of *Edestus* crowns, from 2° to 22°, and is sensitive to specimen size. An individual whorl may include ~5–12 crowns, and represents a relatively short ontogenetic series for the animal. Within a single whorl, insertion angle does not vary much, and small deviations may be attributed to measurement error. Only the Jillson ([20], S1 Figure) specimen shows a significant flattening of curvature along the base of the whorl that cannot be accounted for by taphonomic effects.

Fig 2. Occlusal curvature of *Edestus* tooth whorls.

(A) Symmetric-crowned whorls. (B) Asymmetric-crowned whorls. Upper whorls have solid lines, lower whorls are dashed.

Summing the insertion angle between crowns over the length of a whorl (starting from the anterior, more juvenile teeth) gives a cumulative curvature of the occlusal surface. *Edestus* whorls in this study have occlusal curves that range in radius between 5 and 45 cm. Generally, as crown width (W) increases, spacing between crown apices (D) increases, and so does insertion angle. It follows that juvenile whorls are more tightly curved than their adult counterparts. Further, our limited dataset suggests that

asymmetric-crowned whorls maintain higher curvature at large size compared to symmetric-crowned whorls (e.g., FMNH PF 2317 versus ANSP 22393). The holotype *E. giganteus* (AMNH 225) is an outlier in having the largest teeth and the most gently curved whorl (45 cm radius) of any specimen.

Given that occlusal curvature varies with size, and the number of complete whorls to measure is relatively small, insertion angle and its aggregate, the occlusal curve, are unlikely to be useful quantitative taxobases to distinguish *Edestus* species at this time.

Linear measures of teeth

Crown and root proportions of *Edestus* teeth distinguish symmetric versus asymmetric specimens (Table 2). Specifically, the height to width ratio (H:W) of the tooth crown (Fig 3) shows distinct populations for each basic form. Asymmetric crowns are the taller of the two forms, and follow a linear trend ($r^2 = 0.89$) with a mean H:W of 1.08. The ratio of root length to crown width has a mean of 3.8 for the asymmetric group, and follows a linear trend with size. Variation in root proportions for a given tooth size can be attributed to incomplete growth in the root. For both ratios, the asymmetric crowns of the holotype *E. triserratus*, *E. mirus*, *E. pringlei*, *E. minusculus*, *E. minor*, and *E. kolomnensis* fit closely within the population of FMNH specimens.

Fig 3. Crown dimensions for all studied *Edestus* specimens.

Symbology corresponds to revised species designations. Holotype specimen of *Edestus* species are marked with vertical line, see Table 1 for specimen identification.

Table 2. Tooth metrics for *Edestus* specimens.

Specimen	Elem.	Posn.	Type	Crown W	Crown H	H:W	Root L
Edestus heinrichi							
ANSP 22376	t			3.13	1.75	0.56	18.46
ANSP 22378	t			2.35	1.24	0.53	0.00
ANSP 22379	t			3.71	2.29	0.62	0.00
ANSP 22380	t			2.12	1.75	0.83	0.00
ANSP 22381	t			2.60	1.48	0.57	0.00
ANSP 22382	t			3.13	1.35	0.43	0.00
ANSP 22383	t			3.08	1.23	0.40	0.00
ANSP 22384	t			3.59	1.86	0.52	0.00
ANSP 22387	t			2.18	1.15	0.53	0.00
ANSP 22390	t			3.20	1.88	0.59	0.00
ANSP 22398	t			2.61	1.68	0.64	0.00
ANSP 22399	t			3.67	1.74	0.47	0.00
ANSP 22400	t			3.98	1.75	0.44	0.00
ANSP 22401	t			3.34	1.61	0.48	0.00
ANSP 22402	t			2.42	1.49	0.61	0.00
ANSP 22403	t			3.02	1.67	0.55	0.00
ANSP 22404	t			3.12	1.50	0.48	0.00
ANSP 22405	t			2.39	1.28	0.54	0.00
ANSP 22406	t			1.21	0.94	0.77	0.00
ANSP 22412	t			2.40	1.46	0.61	10.45
ANSP 22413	t			1.97	1.07	0.54	9.40
ANSP 22414	t			2.79	1.28	0.46	0.00
ANSP 22415	t			1.79	1.12	0.63	0.00
ANSP 22416	t			3.25	1.59	0.49	0.00
ANSP 22417	t			3.75	1.88	0.50	0.00
ANSP 22418	t			2.38	1.35	0.57	0.00
ANSP 22419	t			3.68	1.53	0.42	0.00
ANSP 22420	t			1.77	0.93	0.53	0.00
ANSP 22423	t			3.14	1.41	0.45	0.00
ANSP 22424	t			2.81	1.26	0.45	0.00
ANSP 22425	t			1.27	0.63	0.50	0.00
ANSP 22426	t			1.65	1.57	0.95	0.00
ANSP 22428	t			2.48	1.76	0.71	0.00

ANSP	22430	t		2.73	1.66	0.61	0.00
ANSP	22431	t		2.28	0.88	0.39	0.00
ANSP	22433	t		3.09	1.96	0.63	0.00
ANSP	22435	t		2.15	0.86	0.40	0.00
ANSP	22436	t		2.56	1.20	0.47	0.00
FMNH	Barret #6	t		0.92	0.78	0.84	7.63
FMNH	C67 Dr 15-4	t		1.74	0.91	0.52	8.19
FMNH	CompPenn#17	t		0.00	0.00	0.00	8.61
FMNH	HQ114	t		1.51	1.11	0.74	0.00
FMNH	HQ1329 B-1	t		2.62	1.78	0.68	13.11
FMNH	HQ1344-B-2	t		2.62	1.46	0.56	11.57
FMNH	HQ46	t		1.53	1.10	0.72	10.81
FMNH	HQ554 A-2	t		1.04	0.59	0.57	4.89
FMNH	HQ555 B-1	t		1.15	0.72	0.63	6.44
FMNH	HQ610 A-3	t		1.27	0.78	0.61	7.00
FMNH	HQ66	t		2.41	0.97	0.41	0.00
FMNH	HQ69,70(B)	t		0.64	0.28	0.43	3.42
FMNH	HQ799 B-1	t		2.77	1.36	0.49	0.00
FMNH	HQ983 A-2	t		1.54	0.83	0.54	6.07
FMNH	Jelliff#2	t		3.02	1.49	0.49	0.00
FMNH	L238	t		1.70	0.78	0.46	8.06
FMNH	L250	t		2.12	1.19	0.56	8.50
FMNH	L309 (J864)	t		1.68	0.85	0.51	7.98
FMNH	L310 (J629)	t		0.59	0.44	0.76	2.99
FMNH	L310 (J859)	t		0.37	0.27	0.73	1.75
FMNH	L311 (J1068)	t		2.57	1.42	0.55	11.26
FMNH	L311 (J288)	t		1.65	0.93	0.56	6.91
FMNH	L311 (J938)	t		1.93	1.01	0.52	8.01
FMNH	L312(J61)	t		1.54	1.00	0.65	7.20
FMNH	L314	t		1.75	1.25	0.71	7.97
FMNH	L315A(PF2320)	t		2.28	1.28	0.56	10.11
FMNH	L316	t		2.38	1.66	0.70	0.00
FMNH	L317(2314)	t		0.55	0.60	1.09	4.31
FMNH	Logan 115 (J657)	t		1.14	0.96	0.84	6.25
FMNH	MontgomeryCk dr5	t		2.04	1.30	0.64	9.15
FMNH	MQ236 (PF2852)	t		1.98	1.65	0.84	0.00
FMNH	PF2320	t		2.40	1.56	0.65	10.22
FMNH	PF-2323	t		1.60	0.93	0.58	7.27
FMNH	PF-2330	t		0.53	0.32	0.61	1.82
FMNH	PF2331 J-122	t		2.32	1.44	0.62	6.98
FMNH	PF2335 J288	t		2.01	1.10	0.55	6.83
FMNH	PF-2339	t		1.16	0.69	0.60	0.00
FMNH	PF-2341	t		0.63	0.49	0.79	2.38
FMNH	PF-8049	t		2.12	1.62	0.76	0.00
FMNH	PF-8404	t		3.14	1.60	0.51	0.00
FMNH	Pit 14(17&18)	t		2.45	1.03	0.42	0.00
FMNH	Pit 14(Peabody Coal)	t		2.27	1.11	0.49	0.00
FMNH	Q208	t		1.26	0.76	0.60	6.39
FMNH	UC3316	t		3.02	1.47	0.49	0.00
ANSP	22377	t	l	2.71	1.38	0.51	17.39

ANSP	22411	t	l	2.97	1.16	0.39	11.67
FMNH	C67 Dr 15-1	t	l	2.18	0.98	0.45	13.05
FMNH	C67 Dr 15-3	t	l	2.33	1.11	0.47	13.07
FMNH	CompPenn#19	t	l	1.63	1.00	0.61	11.70
FMNH	Fish pit 12	t	l	1.67	0.95	0.57	11.79
FMNH	HQ1374 A-3	t	l	2.31	1.26	0.55	14.27
FMNH	PF-1024	t	l	2.00	1.02	0.51	10.56
FMNH	X-Ray #10	t	l	2.01	0.89	0.44	11.21
ANSP	22391 .1	w	u	2.33	1.61	0.69	12.39
ANSP	22391 .2	w	u	2.34	1.55	0.66	12.33
ANSP	22391 .3	w	u	2.23	1.67	0.75	0.00
ANSP	22391 .4	w	u	2.22	1.63	0.73	0.00
ANSP	22391 .5	w	u	2.08	1.63	0.79	0.00
ANSP	22391 .6	w	u	2.27	1.69	0.74	0.00
ANSP	22392 J-3 .1	w	u	3.23	1.89	0.58	17.58
ANSP	22392 J-3 .2	w	u	3.25	1.96	0.60	17.35
ANSP	22392 J-3 .3	w	u	3.04	1.81	0.60	15.84
ANSP	22392 J-3 .4	w	u	3.14	1.87	0.60	14.37
ANSP	22392 J-3 .5	w	u	2.88	1.79	0.62	12.52
ANSP	22392 J-3 .6	w	u	3.03	1.95	0.64	0.00
ANSP	22392 J-3 .7	w	u	2.86	1.75	0.61	0.00
ANSP	22392 J-3 .8	w	u	2.69	1.71	0.64	0.00
ANSP	22396 J-1 .1	w	u	3.01	1.51	0.50	19.41
ANSP	22396 J-1 .2	w	u	3.08	1.76	0.57	19.37
ANSP	22396 J-1 .3	w	u	2.82	1.61	0.57	19.40
ANSP	22396 J-1 .4	w	u	3.26	1.77	0.54	19.50
ANSP	22396 J-1 .5	w	u	3.10	1.76	0.57	0.00
ANSP	22396 J-1 .6	w	u	3.03	1.83	0.60	0.00
ANSP	22396 J-1 .7	w	u	3.05	1.81	0.59	0.00
ANSP	22396 J-1 .8	w	u	2.80	1.86	0.66	0.00
ANSP	22396 J-1 .9	w	u	2.87	1.79	0.62	0.00
ANSP	22396 J-1 .10	w	u	2.64	1.78	0.68	0.00
ANSP	22396 J-1 .11	w	u	2.62	0.00	0.00	0.00
FMNH	PF-2845	w	u	0.44	0.37	0.84	0.00
FMNH	PF-8735 .1	w	u	0.00	0.00	0.00	20.20
FMNH	PF-8735 .2	w	u	3.50	2.00	0.57	19.20
FMNH	PF-8735 .3	w	u	3.30	1.80	0.55	19.00
FMNH	PF-8735 .4	w	u	3.40	2.00	0.59	17.70
FMNH	PF-8735 .5	w	u	3.30	1.90	0.58	15.70
FMNH	PF-8735 .6	w	u	3.30	2.00	0.61	0.00
FMNH	PF-8735 .7	w	u	3.40	0.00	0.00	0.00
FMNH	PF-8735 .8	w	u	3.20	2.10	0.66	0.00
FMNH	PF-8735 .9	w	u	3.20	0.00	0.00	0.00
FMNH	UF30 .1	w	u	3.10	1.80	0.58	16.90
FMNH	UF30 .2	w	u	3.00	0.00	0.00	16.60
FMNH	UF30 .3	w	u	3.00	0.00	0.00	16.50
FMNH	UF30 .4	w	u	3.00	0.00	0.00	15.80
FMNH	UF30 .5	w	u	2.90	0.00	0.00	14.50
FMNH	UF30 .6	w	u	2.90	0.00	0.00	0.00
FMNH	UF30 .7	w	u	2.90	0.00	0.00	0.00

FMNH	UF30 .8	w	u		2.80	2.10	0.75	0.00
FMNH	UF30 .9	w	u		2.80	2.10	0.75	0.00
FMNH	HeslerQ 26	w	l		1.52	0.81	0.53	7.13
ANSP	22373 J-5 .1	w	l		3.25	1.73	0.53	21.65
ANSP	22373 J-5 .2	w	l		3.48	1.68	0.48	21.33
ANSP	22393 J-4 .1	w	l		3.28	1.84	0.56	22.09
ANSP	22393 J-4 .2	w	l		3.29	1.83	0.56	0.00
ANSP	22393 J-4 .3	w	l		3.21	1.80	0.56	0.00
ANSP	22393 J-4 .4	w	l		3.38	1.86	0.55	0.00
ANSP	22393 J-4 .5	w	l		3.31	1.91	0.58	0.00
ANSP	22393 J-4 .6	w	l		3.41	1.91	0.56	0.00
ANSP	22393 J-4 .7	w	l		3.07	2.04	0.66	0.00
ANSP	22393 J-4 .8	w	l		3.20	1.95	0.61	0.00
ANSP	22393 J-4 .9	w	l		3.25	2.27	0.70	0.00
ANSP	22393 J-4 .10	w	l		3.11	1.84	0.59	0.00
ANSP	22393 J-4 .11	w	l		3.00	2.11	0.70	0.00
ANSP	22393 J-4 .12	w	l		3.14	1.84	0.59	0.00
PIN	RAN 1988/1	t			2.87	2.30	0.80	13.78
AMNH	488 cast	w	u	hein	2.98	2.03	0.68	16.55
USNM	V 182450 .1	w	u	cren	3.18	0.00	0.00	16.48
USNM	V 182450 .2	w	u	cren	2.80	0.00	0.00	15.73
USNM	V 182450 .3	w	u	cren	2.84	1.64	0.58	14.80
USNM	V 182450 .4	w	u	cren	2.70	1.74	0.64	13.71
USNM	V 182450 .5	w	u	cren	2.85	0.00	0.00	0.00
USNM	V 182450 .6	w	u	cren	2.58	1.58	0.61	0.00
USNM	6049 .2	w	u	serr	2.62	1.93	0.73	12.18
USNM	6049 .3	w	u	serr	2.46	2.00	0.81	11.40
USNM	6049 .4	w	u	serr	2.46	1.92	0.78	9.71
USNM	6049 .5	w	u	serr	2.29	1.86	0.81	0.00
USNM	6049 .6	w	u	serr	2.25	0.00	0.00	0.00
PIN	RAN 1988/2	t		karp	2.90	2.15	0.74	14.00
n/a	Trautschold [14]	t		proto	3.00	2.20	0.00	0.00
Edestus vorax								
ANSP	9899	w		vorax	4.46	4.16	0.93	11.97
AMNH	225	w		gigan	6.85	8.36	1.22	29.15
KGS	Greb et al. 2011 [21]	W			4.76	4.03	0.85	21.65

Specimen		Elem	Posn	Type	Crown W	Crown H	Crown H:W	Crown uw	Crown uh	Crown uw:uh	Root L
Edestus minor											
AMNH	FF 477	t		minor	1.83	2.51	1.37	1.22	1.58	0.77	
USNM	7255 .2 low	w	l	mirus	2.24	2.47	1.10	1.35	1.63	0.83	
USNM	7255 .3 low	w	l	mirus	2.03	2.55	1.26	1.40	1.77	0.79	
USNM	7255 .4 low	w	l	mirus	2.00	2.57	1.29	1.59	1.83	0.87	
USNM	7255 .5 low	w	l	mirus	2.20	2.67	1.21	1.40	1.62	0.86	

USNM	7255 .2 up	w	u	mirus	2.59	2.45	0.95	1.63	1.82	0.90	
USNM	7255 .3 up	w	u	mirus	2.61	2.74	1.05	1.66	2.02	0.82	10.00
USNM	7255 .4 up	w	u	mirus	2.71	2.54	0.94	1.71	1.81	0.94	
USNM	7255 .5 up	w	u	mirus	2.68	2.92	1.09	1.80	2.05	0.88	
GSM	49368	t		pring	1.26	1.56	1.24	0.90	1.18	0.76	3.89
TMM	40234-8	t			2.83	2.60	0.92	1.53	1.58	0.97	
FMNH	HQ42	t			1.97	1.68	0.85	1.42	1.61	0.88	5.71
FMNH	HQ435 B4	t			2.63	2.57	0.98	1.54	1.96	0.79	
FMNH	HQ65	t			0.73	0.80	1.10	0.51	0.72	0.71	3.07

Edestus triserratus

GSM	31410	t		triser	1.83	1.93	1.05	0.99	1.45	0.68	5.90
TSNiGR	11/1865	t		minusc	1.35	1.40	1.04	0.59	1.23	0.48	
PIN	RAN2804/726	t		kolomn	3.45	3.80	1.10	2.16	2.66	0.81	
PIN	RAN2804/511	t			0.86	1.08	1.26	0.46	0.87	0.53	3.37
AMNH	485	w	l		3.04	3.36	1.11	2.05	2.72	0.75	11.86
n/a	Jillson [20]	w	u		3.71	3.19	0.86	1.84	2.56	0.72	14.58
CAS	n/a	w	u		2.93	3.27	1.12	1.95	2.47	0.79	14.98
FMNH	L309J865	t			2.73	2.64	0.97	1.64	2.48	0.66	9.07
FMNH	L309J872	t			1.56	2.22	1.42	1.16	2.03	0.57	7.59
FMNH	L310J870	t			1.86	1.97	1.06	1.10	1.80	0.61	
FMNH	L311J23	t			1.63	1.72	1.06	1.17	1.60	0.73	
FMNH	L312J852	t			1.67	2.16	1.29	1.13	2.03	0.56	8.78
FMNH	PF2317.1	w	l		2.72	2.40	0.88	1.60	1.97	0.81	9.50
FMNH	PF2317.2	w	l		2.47	2.40	0.97	1.40	1.89	0.74	9.80
FMNH	PF2317.3	w	l		2.69	2.51	0.93	1.68	1.98	0.85	
FMNH	PF2317.4	w	l		2.74	2.59	0.95	1.65	2.12	0.78	
FMNH	PF2317.5	w	l		2.60	2.49	0.96	1.60	2.15	0.74	
FMNH	PF2317.6	w	l		2.67	2.53	0.95	1.66	2.10	0.79	
FMNH	PF2317.7	w	l		2.48	2.47	1.00	1.68	2.10	0.80	
FMNH	PF2318	t			2.62	2.48	0.95	1.57	2.29	0.69	
FMNH	PF2319.1	t			3.07	3.12	1.02	1.71	2.63	0.65	11.48
FMNH	PF2319.2	t			3.99	3.29	0.82	2.04	2.78	0.73	11.01
FMNH	PF2319.3	t			3.32	3.43	1.03	2.16	2.87	0.75	9.49
FMNH	PF2326	t			2.39	2.45	1.03	1.53	2.31	0.66	
FMNH	PF2336	t			2.91	2.16	0.74	1.49	1.88	0.79	
FMNH	UC2092	t			2.98	2.80	0.94	1.46	2.28	0.64	11.47
FMNH	C67Dr15	t			2.37	2.20	0.93	1.31	1.79	0.73	
FMNH	HQ72	t			2.56	2.45	0.96	1.61	2.15	0.75	8.75
USNM	14790	t			1.53	1.83	1.20	0.94	1.61	0.58	7.62

Teeth within a whorl are measured separately and their position in the whorl is annotated in the specimen identifier with a sequential decimal suffix (*e.g*., .1 is the anterior-most tooth, .2 is the adjacent tooth to the posterior, etc.). Type = Original species diagnoses: hein = *E. heinrichi*; cren = *E. crenulatus*; serr = *E. serratus*; karp = *E. karpinskyi*; kolomn = *E. kolomnensis*; proto = *E. protopirata*; minor = *E. minor*; mirus = *E. mirus*; triser = *E. triserratus*; pring = *E. pringlei*; minusc = *E. minusculus*; vorax = *E. vorax*; gigan = *E. giganteus*. Elem. = Element: t = tooth; w = whorl. Posn = Position: l = lower whorl; u = upper whorl. Crown width (W), crown height (H), root length (L) and root curve in cm.

The population of symmetric crowns, composed mostly of ANSP and FMNH teeth, show a weaker positive correlation with size ($r^2 = 0.45$) and a mean H:W of 0.61, much lower than the asymmetric group. The high variance of H:W is observed even among multiple crowns from the same whorl. For example, 12 crowns measured from ANSP 22393 have H:W ranging between 0.55 and 0.70. The holotypes of *E. crenulatus, E. heinrichi*, *E. protopirata*, *E. serratus*, *E. karpinskyi* plot at or slightly above the mean with H:W consistently <0.80, whereas the very large crowns of the holotypes *E. vorax* and *E. giganteus* plot closer to the asymmetric group mean with H:W > 0.9.

The roots of symmetric-crowned teeth are proportionately longer than the asymmetric group, with a mean root-length to width ratio of 5. This value increases to 6 with the largest teeth (3 cm width). Again, *E. vorax* and *E. giganteus* are the only

holotypes with symmetric crowns that plot as outliers, having short roots only 2.5 to 4 times greater than crown width.

Geometric morphometry of tooth crowns

Geometric analysis of all *Edestus* tooth crowns demonstrates distinct populations for the asymmetric and symmetric specimens (Fig 4). The first principle component captures 88% of crown shape variation, with positive values corresponding to a short, stout, and symmetrical crown. The holotypes of *E. protopirata*, *E. crenulatus*, *E. heinrichi*, *E. serratus*, *E. karpinskyi*, and *E. giganteus* fit in this grouping of positive PC1 space. In negative PC1 space, the apex is deflected anteriorly, and the overall crown is narrow and tall. The asymmetric-crowned holotypes of *E. kolomnensis*, *E. pringlei*, *E. minusculus*, *E. mirus* (both upper and lower whorl teeth) and *E. minor* fit in this population. Given the distinct populations from the analysis of all specimens, the symmetric and asymmetric groups were each analyzed separately.

Fig 4 Geometric morphometry of all *Edestus* specimens.

Type specimens have outline black circle. Holotype *E. mirus* crowns from upper and lower whorls shown in red and blue circles, respectively.

Principle components analysis of the symmetric group results in a similar distribution of shape observed in the combined analysis (Fig 5A). Positive PC1 corresponds to a short, wide crown typified by the holotypes of *E. heinrichi* and *E.*

crenulata, whereas the holotypes of *E. protopirata*, *E. karpinskyi*, and *E. serratus* occupy negative PC1 space. The holotype of *E. giganteus* is an outlier in negative PC1 space, with a very tall crown. Positive PC2 corresponds to an anterior tilted apex, which distinguishes *E. protopirata* from other specimens. Crown width has a small positive correlation with PC1 (r^2=0.25, excluding *E. giganteus* as an outlier) and does not correlate with PC2. Generally, with increased size, teeth are wider and shorter in shape. This observation is documented by consecutive crowns on a single whorl, as illustrated by five ANSP whorl specimens from a mine locality above the Herrin Coal (Fig 5B). Furthermore, crowns from these whorls span 0.5 units of the PC1 axis, and collectively span from -0.1 to +1.0, which includes all holotypes, but the outlier *E. giganteus*.

Fig 5. Geometric morphometry of *Edestus* with symmetric-crowned teeth.
(A) PCA of all crowns color-coded by crown width size to show ontogenetic variation. Holotypes have outlined circle symbol. (B) Five whorls from ANSP provide PC1 shape of consecutive crowns plotted by crown width. Holotypes have outlined circles filled white. Note significant outlier *E. giganteus* holotype above the y-axis excursion.

Analysis of the asymmetric specimens (Fig 6) shows a reorientation of the PCs compared to analysis of all *Edestus* specimens (Fig 4). Positive PC1 (51% variance) corresponds to a stouter crown with an anterior tilt, and *E. kolomnensis* is the only holotype to have this aspect. There is no correspondence between size and PC1 (r^2 = 0.019), and multiple crowns from a single whorl (e.g., FMNH PF2319.1–3) show a broad

range along this axis. PC2, which accounts for 35% variance, distinguishes broad crowns in positive space versus crowns having a slender, bullet shaped outline in negative space. PC2 corresponds with size, and thus has potential to show an ontogenetic series. Plotting crown width against PC2 (Fig 6B) shows a weak positive correlation ($r^2 = 0.16$), i.e., that as teeth grow wider at the base, the upper part of the crown also broadens. The positive correlation may be stronger, however, if considering two distinct size-shape curves are represented in the graph, each resulting in adults with wider versus bullet shaped crowns. The upper grouping includes *E. mirus* and *E. minor* holotypes and *E. pringlei* at smaller size. The lower grouping includes *E. kolomnensis* in the large size, and *E. minusculus* at the smaller size. Seven crowns from a single whorl, FMNH PF2317, plot closest to the lower grouping.

Fig 6. Geometric morphometry of *Edestus* with asymmetric-crowned teeth.

(A) PCA of all crowns color-coded by crown width size to show ontogenetic variation. Multiple crowns from holotype *E. mirus* from both upper and lower whorls outlined. All holotypes have outlined circle symbol. (B) PC2 plotted by crown width. Two populations of crowns separated by dashed line. (C) PC2 of all asymmetric crowns correlates strongly with the upper ratio metric. (D) A meristic approach to distinguishing two asymmetric crown types for *Edestus*, based on geometric ordination in B. The position of *E. triserratus* in the graph based only on meristic measures of the holotype.

The shape described by PC2 is positively correlated (r^2=0.84) to direct measures of the upper crown ratio (upper width by upper height) (Fig 6C). These direct measurements for the asymmetric specimens are plotted by crown width to reveal a similar pattern observed in the PCA: two ontogenetic series differing in the upper crown ratio by tooth width (Fig 6D). The holotype of *E. mirus* is significant in this sample because it illustrates overlapping upper ratios with variance of 0.1 and that lower crowns are 0.5 cm narrower than upper crowns. Although the type of *E. triserratus* is too incomplete for a full series of landmarks, it does preserve the base of the crown up to the inflection points where upper width is measured. Estimation by Newton ([22], Pl I.3) of a complete crown suggests the holotype of *E. triserratus* (Fig 6D) would plot closest to the lower grouping of bullet-shaped asymmetric teeth that also includes holotypes *E. minusculus* and *E. kolomnensis*. Crowns from the straight-based whorls, CAS and Jillson, also plot in the lower grouping.

Summary of taxobases for *Edestus*

Results of geometric and meristic analyses identify crown shape, root length, and qualitative observations of whorl curvature to be useful taxobases for *Edestus* (Fig 7). Four morphological concepts are defined from these taxobases.

Fig 7. Four species of *Edestus*.

(A) *E. heinrichi* crown, AMNH 466G. (B) Xray of two small whorls of *E. heinrichi,* FMNH HQ 492 B-2. (C) *E. minor* crown, GSM 49368. (D) *E. triserratus* tooth, FMNH UC 2092. (E) *E. triserratus* tooth, GSM 31410. Scale bar same for A-E. (F) *E. minor* associated

upper and lower whorls, USNM 7255; (G) Xray of subadult *E. triserratus* associate upper and lower whorls with anterior jaws, FMNH PF 8047. Note two crowns lacking bases forming at posterior of upper whorl (arrow). (H) Adult *E. triserratus* upper whorl, CAS specimen, oriented with (I) similarly-sized lower whorl of *E. triserratus*, AMNH 485. (J) *E. heinrichi* whorl, AMNH 488 cast. (K) *E. heinrichi* whorl, USNM V 182450. (L) *E. heinrichi* whorl, AMNH 6049. (M) Xray of *E. heinrichi* whorl, FMNH HQ 26. (N) *E. heinrichi* tooth, FMNH HQ 1374 A3. (O) *E. vorax*, AMNH 225, note that all crown apices have been repaired. Scale bar same for G-O. Images C, E courtesy of British Geological Survey.

Systematic Paleontology

Class Chondrichthyes Huxley, 1880 [23]

Subclass Euchondrocephali Lund and Grogan, 1997 [24]

Order Eugeneodontiformes Zangerl, 1981 [1]

Superfamily Edestoidea Hay, 1929 [25]

Family Edestidae Jaekel, 1899 [26]

Genus *Edestus* Leidy, 1856 [15]

Type species. *Edestus vorax* Leidy 1856 [15]

Included species. *E. heinrichi, E. minor, E. triserratus, E. vorax*

Diagnosis. Single symphyseal whorl in each upper and lower jaw, dimorphic, forming open spiral of up to 12 teeth, shed anteriorly; tooth crowns laterally compressed,

triangular in shape with slightly concave edges bearing denticles; basal projection of crown directed posteriorly; elongate tooth roots stacked *en echelon* anteriorward along base of whorl opposite the crowns, whorl base has convex bulge.

Occurrence. Early to Middle Pennsylvanian (late Baskirian to Moscovian). Britain, United States, Russia.

Remarks. The species *Lestrodus newtoni* Woodward 1917 [27](Obruchev, 1953 [11]) of the Namurian Millstone Grit Group (middle Morrowan age equivalent [12, 29]) is excluded from *Edestus*. The holotype of *L. newtoni*, GSM 28346, shows no evidence for a convex bulge opposite the tooth crowns on the whorl, a distinct feature common to all upper and lower whorls of *Edestus*. Furthermore, no clear evidence exists to demonstrate that *L. newtoni* had both upper and lower whorls. Three genera are formally synonymized under *Edestus*, following [7], including *Edestes* Miller 1877 [29], *Edestodus* Obruchev 1953 [11], and *Protopirata* Trautschold 1888 [30].

Edestus minor Newberry in Newberry and Worthen, 1866 [31]

Figures 7C, F

Edestus minor Newberry in Newberry and Worthen 1866 [31], pp. 84-85, pl. IV, fig. 24.

Itano 2014 [8], figs. 9-10.

Edestus mirus Hay 1912 [5], p. 31-38, pl. 1-2.

Edestus pringlei Watson 1930 [32], pp. 69-71, fig. 1A.

Edestus minor Itano et al. 2012 [12], fig 12.

Diagnosis. Obtuse triangular crown with gradual taper to apex, anteriorly; denticles fine; roots short in greatly curved lower whorl, moderate roots with slight curve in upper whorl.

Description. Crown height to width ratio near or above 1.0. Upper ratio ranges from 0.7 to 1.0 from juvenile (W<2cm) to adult teeth (W>2cm). The apical angle is approximately 30-35°. In adult crowns, fine denticles are ~1 mm in width and may be subdivided by more than one cusp. USNM 7255 is the most complete example of the species, and demonstrates slight dignathism in size and shape of crowns of the upper and lower whorls. USNM 7255 (Figs 7F, 8) shows five lower crowns and a broken upper whorl with space for at least 6-8 crowns, and therefore may follow an 8:5 ratio for upper and lower whorl teeth. The convex bulge is conspicuous in the lower whorl of USNM 7255, and aligns with the junction of the symphysis of Meckelian cartilage preserved in the specimen. Crown morphology of the type AMNH FF477 most closely resembles an upper whorl tooth, but unfortunately the root is missing.

Fig 8. Illustration of revised *Edestus* species whorls and teeth.

(A) *Edestus minor* represented by associated upper and lower whorl. (B) *Edestus triserratus* adult represented by two separate specimens of compatible size, and subadult represented by associated upper and lower whorls. (C) *Edestus heinrichi* adult form represented by two separate specimens of compatible size, and subadult represented by associated upper and lower whorls. (D) *Edestus vorax* adult upper whorl and lower tooth of separate individual. Scale bar for all specimens.

Holotype. *E. minor*, AMNH FF477, Posey County, Indiana, age unknown;

Other material examined. Six specimens including USNM 7255, GSM 49368, TMM 40234-8, and FMNH specimens HQ42, HQ435 B4, and HQ65.

Occurrence. Late Bashkirian to Moscovian. Westphalian B of Britain, Desmoinesian of U.S.A.

Remarks. The holotype specimen of *E. minor* (AMNH FF477, see [8], fig. 9, 10) is a single crown and lacks most of the root. Newberry [31] described this holotype in the context of a separate 7-crown whorl (ACM 85 and its cast, AMNH 485) first described by Hitchcock [33], noting that the latter was “similar, if not identical” to the holotype. Our geometric analysis demonstrates a fundamental shape difference between the two specimens despite their sizes being comparable. Instead, the holotype *E. minor* most closely resembles an upper whorl crown of USNM 7255, the holotype of *E. mirus*, which therefore becomes a junior synonym of *E. minor*.

Edestus triserratus Newton, 1904 [22]

Figures 7D, E, G, H, I, S1 Figure

part *Edestus vorax* Newberry and Worthen, 1870 [34], pp. 353, pl. 1, fig. 2. Mistakenly identified.

Edestus triserratus Newton, 1904 [22], pp. 3-6, pl. I

Edestus minusculus Hay, 1909 [35], pp. 48-50, fig. 5. *E.* cf. *minor* Karpinsky, 1899 [3], p.12-15, fig. 15-17, Pl. IV, fig. 12a-c, 13.

Edestodus minusculus (Hay 1909 [35]) Obruchev 1953 [11] p. 52, fig. 24. 13 Lebedev, 2001 [13], pl. 44, fig. 4.

Edestodus kolomnensis (Lebedev 2001 [13]), pl. 44, fig. 5.

Holotype. *E. triserratus*, GSM 31410, Westphalian B (upper Bashkirian), Staffordshire, England (Newton, 1904 [22]).

Diagnosis. Obtuse triangular crown narrowing to bullet-shaped apex, anteriorly; denticles fine; root proportionately longer than *Edestus minor*; upper whorl with massive, straight base; lower whorl slender, more tightly curved.

Occurrence. Late Baskirian to Moscovian. Westphalian B of Britain; upper Atokan to Desmoinesian of United States (Colorado, Illinois, Indiana, Iowa, Kentucky, Oklahoma, Michigan, South Dakota, Texas); Myachkovian to ?early Krevyakian of Russia.

Description. Crown height to width ratio near or above 1.0. Upper ratio ranges from 0.5 to 0.8 from juvenile (W<2cm) to adult teeth (W>2cm). The apical angle is approximately 30-35°. In adult crowns, fine denticles are ~1 mm in width and may be subdivided by more than one cusp. Partially articulated cranial material with upper and lower whorls is known from FMNH PF 8047 (Figs 7G, 8). This specimen shows unrooted teeth at the posterior ends of the upper and lower whorls. The tooth count in this specimen shows 10 upper crowns and 6 lower crowns, but only 8 and 5, respectively, are rooted to the whorl. A lower adult whorl (ISM 497337, S1 Figure) similarly shows, at the posterior end

of the whorl, a cluster of crowns most recently formed and have abbreviated roots. The upper whorl of the species, in adult form, is much less curved than its lower counterpart. The CAS specimen is a partly disaggregated whorl, when reconstructed shows very little curvature over the span of 8 crowns, and similarly, the whorl described and figured by Jillson [20] shows a nearly straight 7-crowned whorl of the species.

Other material examined. Includes 23 specimens, TSNiGR 11/1865, DMNH 61071, USNM14790, AMNH 485 (cast of ACM 85 Hitchcock), PIN RAN 2804/726, PIN RAN 2804/511, CAS specimen, Jillson specimen, ISM 497337, and FMNH specimens L309J865, L309J872, L310J870, L311J23, L312J852, PF2317, PF2318, PF2319, PF2326, PF2336, PF8047, UC2092, C67Dr15, and HQ72.

Remarks. Earlier diagnoses of *Edestus* species relied primarily on the number and division of denticles. For example, *E. triserratus* was designated for teeth with trifid denticles, whereas *E. pringlei* is bifid, and *E. minor* has both single and bifid denticles within the same whorl. There exists no accounting of variation within and across species concepts to validate denticle morphology as a discriminating variate. We therefore reject its use as a taxobase, in lieu of more readily quantified attributes presented in this study.

There is sufficient crown material ~~on~~ the holotype of *Edestus triserratus* to determine that the apex of the crown is pointing anteriorly. Both the upper ratio of the

crown and its width can be measured and placed in context of other asymmetric-crowned *Edestus* specimens (Fig 6D).

This species differs from *E. minor* in crown proportions, most notably by being narrower and having subparallel anterior and posterior cutting edges toward the anterior-pointing apex. The posterior edge of the crown is longer and connects to the root at a shallower angle than in *Edestus minor*. This arrangement gives a wider space between individual teeth compared to *E. minor*. Whorls with *E. triserratus* crowns are either slightly curved or nearly straight. Although no articulated specimen of mature *E. triserratus* is yet known, it is reasonable that like other *Edestus* species, the straight whorl resides in the upper jaw and the lower whorl is slightly curved. Except for crown shape, the slightly curved lower whorl of *E. triserratus* and the slightly curved upper whorl of *E. minor* are not distinguishable. The crown arrangement of opposing whorls appear to mesh somewhat like gears near the middle part of the whorl (Fig 8). The degree of overlap (occlusion) is unknown in the species.

Edestus heinrichi Newberry and Worthen, 1870 [34]

Figures 7A, B, J-N

Edestus heinrichi Newberry and Worthen, 1870 [34], pp. 350-353, pl. 1, fig. 1a,b.

Edestus protopirata Trautschold, 1879 [14], p. 49-50, pl. 6, fig. 8. Karpinsky (1899) [3], fig. 6-7.

Protopirata centrodon Trautschold, 1888 [30], p. 49.

Edestus karpinskyi Missuna, 1908 [36], fig. 1, 4.

Edestus crenulatus Hay, 1909 [35], p.43-47, fig. 3-4, pl. 12, fig. 1-3.

Edestus serratus Hay, 1909 [35], p. 47-48, fig. 3-4, pl. 12, fig. 4.

Protopirata protopirata (Trautschold 1879 [14]) Obruchev 1953 [11], Pl.1, fig. 1, Pl. 2, fig. 1, Pl. 4, fig. 2. Lebedev, 2001 [13], pl. 44, fig. 8.

Protopirata karpinskyi (Missuna, 1908 [36]) Obruchev, 1953 [11], Pl. 2, fig 2, Pl 5, fig 2. Lebedev, 2001 [13], pl. 44, fig. 7.

Holotype. *E. heinrichi*, cast AMNH FF 488 (whereabouts of original specimen unknown) Newberry and Worthen (1870) [34].

Diagnosis. Crowns with acute triangular shape, with posterior edge slightly longer than anterior edge, large apical angle; denticles coarse; roots long and straight in upper whorl; slightly curved in lower whorl.

Occurrence. Moscovian. Carbondale Fm of United States (Desmoinesian); Mjachkovski horizon of Russia (Myachkovian to ?early Krevyakian).

Description. Crown height to width ratio is less than 1. Teeth from upper whorls have H:W above average (0.6-0.9) and lower whorl teeth are below average (0.4-0.6). The apical angle is roughly 80-85°. On 3 cm wide crowns, the coarse denticles measure roughly 2 mm in width and may be subdivided by more than one cusp. Partly articulated cranial material from a juvenile, FMNH PF 2204, shows upper and lower whorls in context ([6], figure 1; [2]), with at least 8 upper crowns and 5 lower crowns per whorl. Juvenile tooth roots are longer in the upper whorl and form a more massive whorl in comparison to the slender lower whorl. In larger specimens from the Jeremiah collection, the more massive base of the upper whorl contrasts the slender profile of the

lower whorl. Both whorls have strongly curved occlusal surfaces (Fig 8). The opposite surface, formed by the shingling of V-shaped roots, is nearly straight in the upper whorl, whereas in the lower whorl, it forms a concave curve nearly parallel to the occlusal curve. Refer to [2] for description of a juvenile skull of this species.

Other material examined. Includes 106 specimens, USNM V 182450 (6050), USNM 6049, PIN RAN 1988 /2, Trautschold, 1879 specimen, PIN RAN 1988/1; specimens from ANSP include 22376, 22378, 22379, 22380, 22381, 22382, 22383, 22384, 22387, 22390, 22398, 22399, 22400, 22401, 22402, 22403, 22404, 22405, 22406, 22412, 22413, 22414, 22415, 22416, 22417, 22418, 22419, 22420, 22423, 22424, 22425, 22426, 22428, 22430, 22431, 22433, 22435, 22436, 22377, 22411, 22391, 22392 J-3, 22396 J-1, 22373 J-5, 22393 J-4; specimens from FMNH include Barret #6, C67 Dr 15-4, CompPenn#17, HQ26, HQ114, HQ1329 B-1, HQ1344-B-2, HQ46, HQ554 A-2, HQ555 B-1, HQ610 A-3, HQ66, HQ69,70(B), HQ799 B-1, HQ983 A-2, Jelliff#2, L238, L250, L309 (J864), L310 (J629), L310 (J859), L311 (J1068), L311 (J288), L311 (J938), L312(J61), L314, 315A(PF2320), L316, L317(2314), Logan 115 (J657), MontgomeryCk dr5, MQ236 (PF2852), PF2320, PF-2323, PF-2330, PF2331 J-122, PF2335 J288, PF-2339, PF-2341, PF-8049, PF-8404, Pit 14(17&18), Pit 14(Peabody Coal), Q208, UC3316, C67 Dr 15-1, C67 Dr 15-3, CompPenn#19, Fish pit 12 , HQ1374 A-3, PF-1024, X-Ray #10, PF-2845, PF-8735, UF30 , HeslerQ 26.

Remarks. Teeth in an anterior position on the whorl have longer roots than those at the posterior end, e.g., USNM 6049, ANSP 22392 J3, and FMNH PF 8735. This pattern demonstrates that the posterior root of a tooth continues to grow and results in the anterior advancement of teeth in the whorl. In the course of its travel to an anterior

position, but prior to being ejected, the root stops elongating. The whorl, ANSP 22396 J1, shows this with equivalent lengths of the four anterior teeth. Once fully formed, ejected teeth and anterior teeth of whorls share similar root length proportions. By contrast teeth in posterior positions of the whorl have stunted root proportions and crown dimensions tend to plot higher than average (Fig 3).

Edestus vorax Leidy 1856 [15]

Figure 7O

Edestus vorax Leidy, 1856 [15], pp. 159-160, pl. 15, fig. 1-4.
Edestus giganteus Newberry, 1889 [37], pp. 225-226, pl. XL.

Holotype. *E. vorax*, ANSP 9899 Leidy (1856) [15], Muskogee Co., Oklahoma [10], Desmoinesian.

Diagnosis. Crowns with acute triangular shape, with slightly longer posterior edge, intermediate apical angle; denticles very coarse; roots stout and extend deep below crown.

Occurrence. Moscovian. Desmoinesian of United States.

Other material examined. Includes four specimens, AMNH 225, KGS specimen, FMNH UC 14346, and USNM 330005.

Description. Dimensions are presented in Table 2. Crown height to width ratio greater than 0.9. Denticles wider than 2 mm. Apical angle of roughly 65°.

Materials. Two partial whorls, AMNH 225 and the unpublished KGS specimen [21]; and two broken crowns, FMNH UC 14346 and USNM 330005.

Remarks. The similarity of *E. vorax* and *E. giganteus* was recognized by Branson [10], though subsequent authors have not followed his recommendation for synonymy. Little is known about *E. vorax* due to its sparse fossil record (n = 5), and therefore variation within the species and its ontogeny remain under-sampled. The convex bulge is conspicuous on the KGS and AMNH 225 whorls, hence its inclusion in the genus. Dimorphism in upper and lower whorls is expected, but currently unknown (Fig 8). Itano et al. [12] have suggested that *E. vorax* is a large individual equivalent to *E. heinrichi*. The measurements in this study unequivocally reject this hypothesis. Ontogenetic series of *E. heinrichi* crown height, for example, follows a trajectory (Fig 3), that steepens at roughly 2.3 cm, far short of *E. vorax* crowns known to be beyond 8 cm tall. One could still argue that juvenile *E. vorax* with smaller crowns must exist, yet the *E. heinrichi* population studied here cannot account for short, greatly thickened root structures found in all *E. vorax*. As *E. heinrichi* whorls get larger during ontogeny, their roots consistently elongate posteriorly relative to their crowns with no evidence for a shift toward shortening and thickening with increased crown size. Growth trends documented here strongly support *E. vorax* as a separate species.

Discussion

The largest dataset yet compiled of *Edestus* teeth provides a quantitative foundation for defining species concepts that incorporate ontogeny and disparities

associated with upper and lower dentitions. As in other Paleozoic chondrichthyans, we find species diversity to be substantially decreased when morphometric methods are applied to larger datasets. The four distinct morphological species defined here provide new constraints on the morphology, size, and geographic history of *Edestus* when placed in context of the recently described cranium and jaw of a juvenile *Edestus heinrichi*, FMNH PF 2204 [2].

Analysis of *E. heinrichi* (FMNH PF 2204) demonstrated that the opposing tooth whorls functioned as grasping and slicing tools. As the lower jaw closes toward the fixed upper whorl, it moves anterodorsally, causing teeth to slice for up to three tooth-lengths through the prey item. Upper and lower teeth do not shear past one another (in a true scissor-like motion), but rather stop short and remain parallel during the bite sequence. Given the similar shape of the whorls through ontogeny, it is parsimonious to expect similar jaw mechanics in the adult *E. heinrichi*.

Furthermore, extrapolating the anatomy and joints of the *E. heinrichi* jaw to the other three species is regarded here as reasonable, with some consideration. The asymmetric species *E. triserratus* and *E. minor* have whorls that exhibit a greater degree of curvature, leading one author to imagine the whorls to curl outside the mouth [8]. Using the jaw of *E. heinrichi* as a conservative model, whorls of the asymmetric species, when scaled for size, fit easily within the supporting palatine and Meckel’s cartilages. We might anticipate a shortening of the lower jaw in *E. minor* to take advantage of the greater occlusal curvature to generate more slicing action, as found in the short jaw of *Helicoprion* [38]. *E. vorax* has a substantially more robust tooth whorl and would necessitate an equally robust jaw structure to accommodate its greater size.

Beyond variations in proportions, we regard the jaw morphology observed in *E. heinrichi* (FMNH PF 2204) as conservative within the genus, pending new discoveries of *Edestus* cranial material.

FMNH PF 2204 also provides some rough estimation for maximum body size in *E. heinrichi*. The upper and lower whorls of FMNH PF 2204 measure 10.4 cm and 8 cm, respectively, and are contained within a cranium roughly 25 cm in length. The largest individual *E. heinrichi* whorls include an upper whorl measuring 32 cm (ANSP 22396) and a lower whorl measuring 43 cm (ANSP 22393). Assuming isometric growth, which is a conservative underestimation given the positive allometry found in *Helicoprion* [9], the cranium of *Edestus heinrichi* attained minimum lengths of 77 to 134 cm for these two specimens, respectively. Using a conservative 5:1 body to head length ratio allows a rough estimate of *E. heinrichi* body length of 6.7 m.

The consolidation of species provides some insight on the timing and geographic expansion of *Edestus*. The history of the group is recorded in paleoequatorial to tropical latitudes (Fig 9). Transgressive marine shale deposits overlying coal swamps are the most frequent depositional setting for *Edestus*, with relatively few reported from basinal marine limestones. *Lestrodus newtoni*, whose root and crown morphology bears closest resemblance to *Edestus*, precedes it in Britain during the Namurian regional stage (middle Bashkirian, early Pennsylvanian). The first *Edestus* species have asymmetric crowns and include both *E. minor* and *E. triserratus* in the Westphalian B (latest Bashkirian) coastal marine deposits of England. By Moscovian time, uplift associated with the Variscan orogeny shifts much of Britain's depositional environments to alluvial facies [39], and no further *Edestus* fossils are found in the region. Elsewhere, *Edestus*

diversify and expand their range to include the Russian Platform and central United States coincident with the global Moscovian transgression. *Edestus heinrichi* and *E. triserratus*, as revised here, are found in deposits of regional Myachkovian age, whereas all four *Edestus* species are found in Atokan to Desmoinesian marginal marine deposits in the US. No *Edestus* are reported after the Desmoinesian, suggesting that their stratigraphic range is from late Bashkirian to Moscovian, approximately 313 to 307 Ma.

Fig 9. *Lestrodus* and *Edestus* in space and time.

Paleogeographic map for the Middle Pennsylvanian modified from [40] and sea level curve modified from [41]. Uncertainty in species range shown with dashed boxes. Abbreviations: Kasim. = Kasimovian; *vorax* = *Edestus vorax*; *hein*. = *Edestus heinrichi*; *minor* = *Edestus minor*; *tri.* = *Edestus triserratus*.

Acknowledgements

We are grateful for the enthusiasm shared by R. Troll for these fossils and for introducing us to Zangerl's collection and correspondence. ISU undergraduate, N. Corder, helped measure tooth xrays from the FMNH collections. We thank our museum colleagues for providing access, images, and loans for this project: A. Gishlick (AMNH), W. Simpson (FMNH), D. Bohaska (NMNH), P. Shepherd (British Geological Survey), T.

Daeschler (ANSP), O. Lebedev (PIN), D. Roberts, C. Widga and D. Roberts (CAS), and K. Wellspring (ACM). This research was partly conducted during sabbatical release time funded by Idaho State University.

References

1. Zangerl R. Chondrichthyes I. Paleozoic Elasmobranchii. Handbook of Paleoichthyology, 1981; 3A, Gustav Fischer, New York, 113 p.
2. Tapanila L, Pruitt J, Wilga CD, Pradel A. Saws, scissors and sharks: Late Paleozoic experimentation with symphyseal dentition. Anat Rec, 2018; doi: 10.1002/ar.24046.
3. Karpinsky AP. Über die Reste von Edestiden und die neue Gattung *Helicoprion*. Verh Russisch-Kaiserlichen Mineralogischen Gesellschaft, 1899; 36:361–475.
4. Tapanila L, Pruitt J, Pradel A, Wilga CD, Ramsay JB, Schlader R, Didier DA. Jaws for a spiral-tooth whorl: CT images reveal novel adaptation and phylogeny in fossil *Helicoprion*. Biol Letters, 2013; 9:20130057.
5. Hay OP. On an important specimen of *Edestus*; with description of a new species, *Edestus mirus*. Proc US Nat Mus. 1912; 42:31–38.
6. Zangerl R, Jeremiah C. Notes on the tooth "saw blades" of *Edestus*, a late Paleozoic chondrichthyan. Mosasaur, 2004; **7**:9–18.
7. Ginter M, Hampe O, Duffin C J. Chondrichthyes. Paleozoic Elasmobranchii: Teeth. Handbook of Paleoichthyology, 2010; v. 3D: Munich, Verlag Dr. Friedrich Pfeil, 168 p.

8. Itano WM. *Edestus*, the strangest shark? First report from New Mexico, North American paleobiogeography, and a new hypothesis on its method of predation. Mountain Geologist, 2014; 5:201–221.

9. Tapanila L, Pruitt J. Unraveling species concepts for the *Helicoprion* tooth whorl. J Paleontol, 2013; 87:965–983.

10. Branson CC. Type species of *Edestus* Leidy. Oklahoma Geology Notes, 1963; 23: 275–280.

11. Obruchev DV. Edestid studies and the works by A. P. Karpinsky. Trudy Paleontologicheskogo Instituta Akademii Nauk SSSR, 1953; 45:1–85.

12. Itano WM, Houck KJ, Lockley MG. Systematics and occurrences of *Edestus* (Chondrichthyes) worldwide and new occurrences from Colorado and Texas. Hist Biol. 2012; 24: 397–410.

13. Lebedev OA. Chapter 14. Pozvonochnye, *in* Makhlina MK, Alekseev AS, Goreva AS, Gorjunova RV. et al., eds., Srednij karbon Moskovskoy sineklizy (yuzhnaya chast') Tom 2. Nauchnyi Mir, Moscow, 2001; 196–201.

14. Trautschold H. Die Kalkbrüche von Mjatschkowa. Eine Monographie des oberen Bergkalks. Nouveaux Mémoires de la Société Impériale des Naturalistes de Moscou, 1879; 14:1–82.

15. Leidy J. Descriptions of some remains of fishes from the Carboniferous and Devonian formations of the United States. J Acad Nat Sci Philadel. 1856; 3:159–165.

16. Zangerl R, Richardson ES. The paleoecological history of two Pennsylvanian black shales. Fieldiana Geol Memoir, 1963; 4:1–352.

17. Koenig JW. Midcontinent Pennsylvanian Edestidae. Report of Investigations - Missouri Geol Surv Water Resources, 1965; 33:1–25.

18. Richards BC. Current status of the international Carboniferous time scale, *in* Lucas, S.G., et al., eds., The Carboniferous-Permian Transition. New Mexico Mus Nat Hist Sci Bull. 2013; 60:348–353.

19. ICZN. International Code of Zoological Nomenclature. 1999; Fourth edition. London, U.K. [available online at http://www.iczn.org/iczn/index.jsp]. International Trust for Zoological Nomenclature.

20. Jillson WR. *Edestus minor*: an extinct shark from the Allegheny Series of western Kentucky. 1949; Roberts Printing Co., Frankfort, KY, 16 p.

21. Greb S, Weisenfluh J, Ettensohn F, Ciampaglio C. *Edestus giganteus* shark fossils from the Carbondale Formation (Middle Pennsylvanian, Desmoinesian), western Kentucky, Illinois Basin. Abstract for the 2011 annual meeting of the Kentucky Academy of Science, 2011; Murray State University, Murray (KY).

22. Newton ET. On the occurrence of *Edestus* in the Coal-Measures of Britain. Q J Geol Soc London, 1904; 60:1–9.

23. Huxley TH. On the application of the laws of evolution to the arrangement of the Vertebrata and more particularly the Mammalia. Proc Zool Soc London. 1880; p. 649–662.

24. Lund R, Grogan ED. Relationships of the Chimaeriformes and the basal radiation of the Chondrichthyes. Rev Fish Biol Fisher. 1997; 7:65–123.

25. Hay OP. Second bibliography and catalogue of the fossil Vertebrata of North America. 1929; Volume 1. Carnegie Institution, Washington (DC).

26. Jaekel O. Über die Organisation der Petalodonten. Z Dtsch Geol Ges. 1899; 51:258–298.
27. Woodward AS. On a new species of *Edestus* from the Upper Carboniferous of Yorkshire. Q J Geol Soc London, 1917; 72:1–6.
28. Itano WM. A tooth of *Edestus* from the early Pennsylvanian of Cheshire, UK. Proc Yorkshire Geol Soc. 2013; 59:187–194.
29. Miller S. The American Palaeozoic fossils: a catalogue of the genera and species, with names of authors, dates, places of publication, etc. 1877; Published by the author. Cincinnati.
30. Trautschold H. Ueber *Edestus protopirata* Trd. Zeitschrift der Deutschen Geologischen Gesellschaft, 1888; 40:750–753.
31. Newberry JS, Worthen AH. Descriptions of new species of vertebrates, mainly from the Sub-Carboniferous limestone and Coal Measures of Illinois. Geol Surv Illinois, 1866; 2:9–134.
32. Watson DMS. *Edestus pringlei* sp. nov. Summary of Progress of the Geological Survey of Great Britain and the Museum of Practical Geology for the Year 1929. 1930; Part II, p. 69–73.
33. Hitchcock E. Account of the discovery of the fossil jaw of an extinct family of sharks, from the Coal Formation. Proc Amer Assoc Adv Sci. 1856; 9:229–230.
34. Newberry JS, Worthen AH. Descriptions of vertebrates. Geol Surv Illinois, 1870; 4:343–374.
35. Hay OP. On the nature of *Edestus* and related genera, with descriptions of one new genus and three new species. Proc US Nat Mus. 1909; 37:43–61.

36. Missuna A. Ueber eine neue *Edestus*-Art aus den Karbon-Ablagerungen der Umgebungen von Kolomna. Bull Soc Impér Natural Moscou. 1908; 21:529–535.
37. Newberry JS. The Paleozoic fishes of North America. Monogr US Geol Surv. 1889; 16:1–340.
38. Ramsay JB, Wilga CD, Tapanila L, Pruitt J, Pradel A, Schlader R, Didier DA. Eating with a saw for a saw: functional morphology of the jaws and tooth-whorl in *Helicoprion davisii*. J Morphol. 2015; 276:47–64.
39. Waters CN, Waters RA, Barclay WJ, Davies JR. A lithostratigraphical framework for the Carboniferous successions of southern Great Britain (Onshore). Brit Geol Surv Res Report, 2009; RR/09/01.
40. Wright et al. Towards community-driven paleogeographic reconstructions: integrating open-access paleogeographic and paleobiology data with plate tectonics. Biogeosciences 2013; 10:1529-1541; doi:10.5194/bg-10-1529-2013.
41. Ross CA, Ross JRP. Late Paleozoic sea levels and depositional sequences. Cushman Foundation for Foraminiferal Research, Special Publication 1986; 24:137-149.

Supporting Information

S1 Figure. Line drawings of *Edestus triserratus* whorls. (A) ISM 497337, lower whorl. (B) Jillson specimen, upper whorl.

S2 Appendix. Spreadsheet for principle components analysis for symmetric crowns depicted in Fig 5A.

S3 Appendix. Spreadsheet for principle components analysis for asymmetric crowns depicted in Fig 6A.

Fig 1

Click here to download Figure FIG1 Terminology.eps

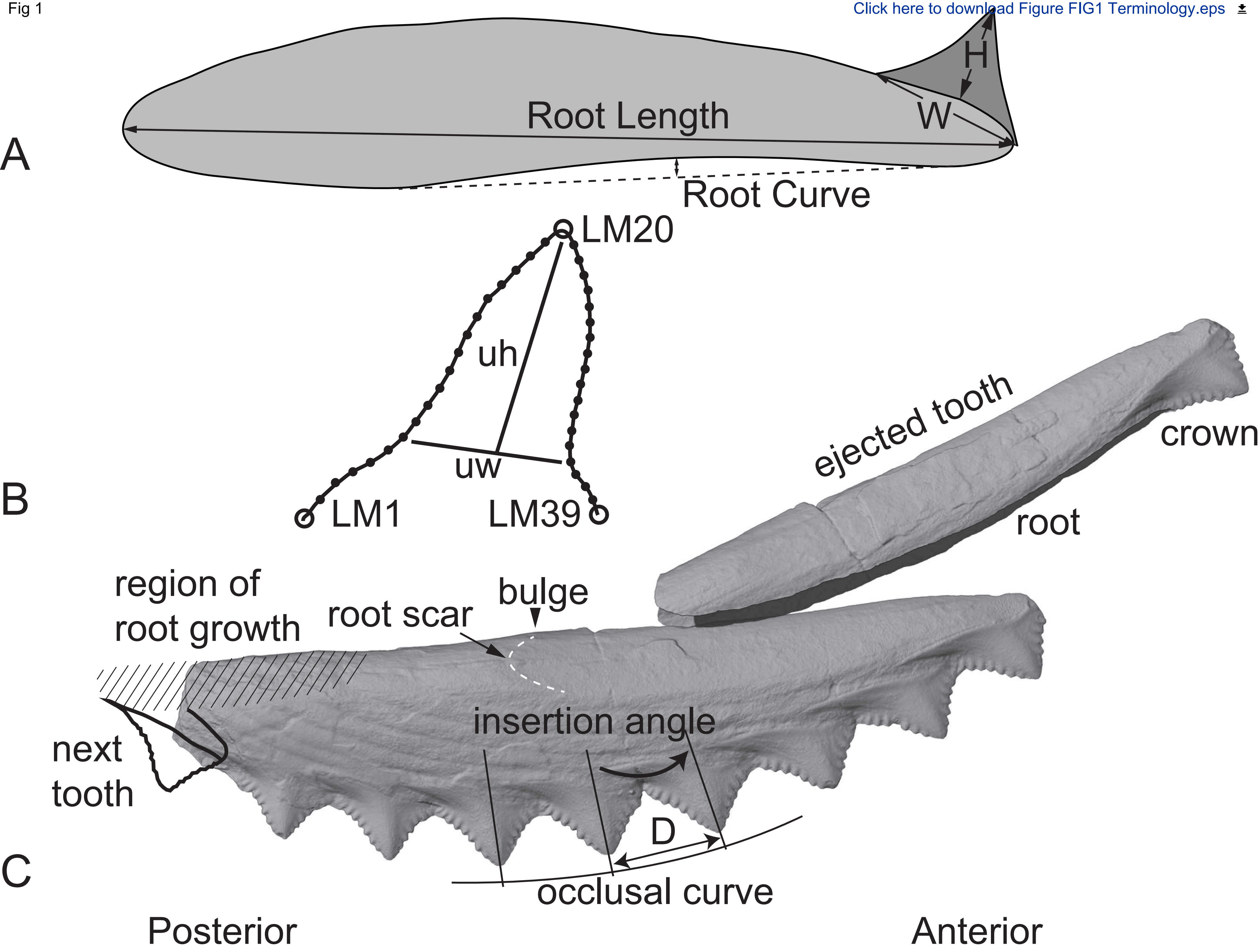

Fig 2

Click here to download Figure FIG2 Spiral Geometry.eps

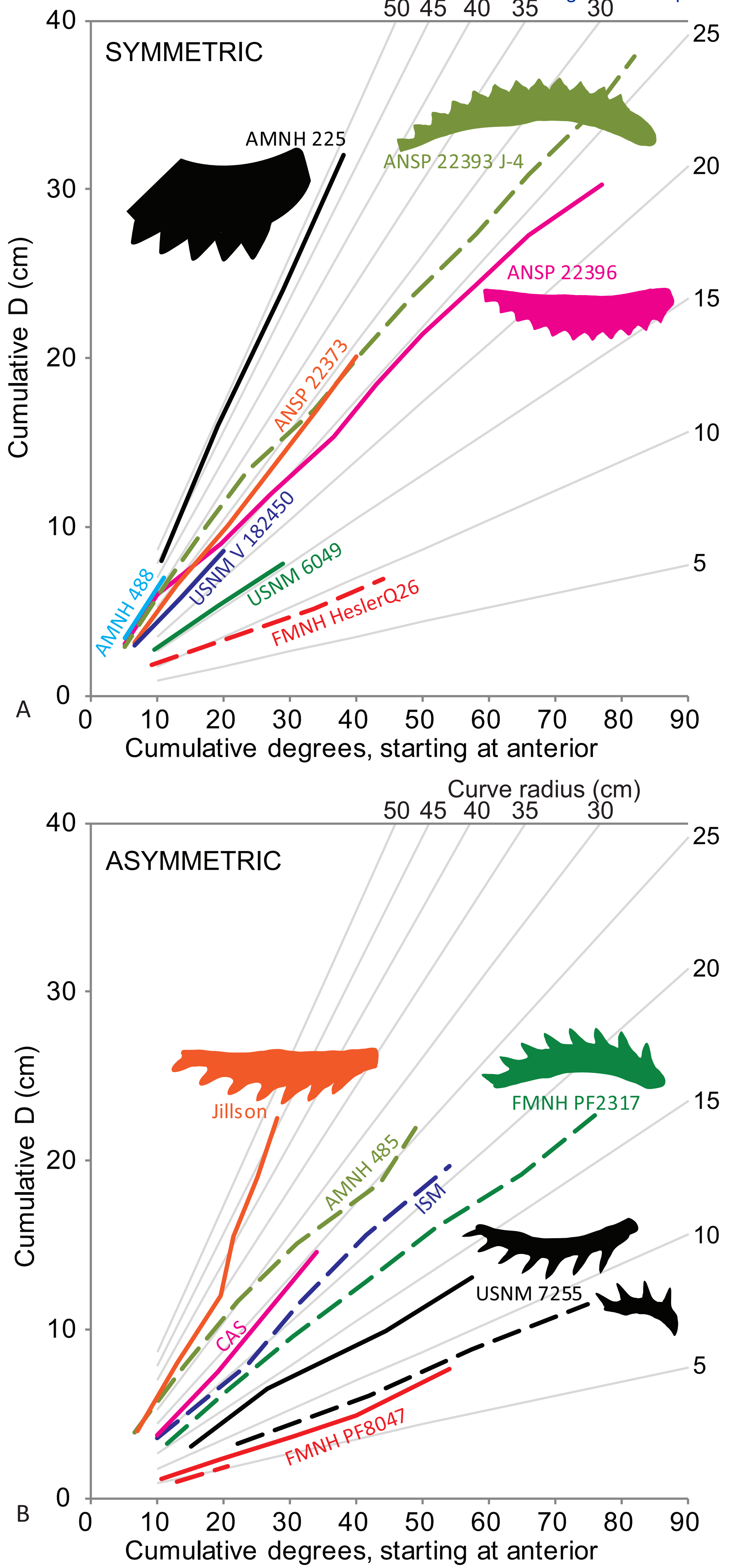

Fig 3

Click here to download Figure FIG3 All-Edestus spp -- new.eps

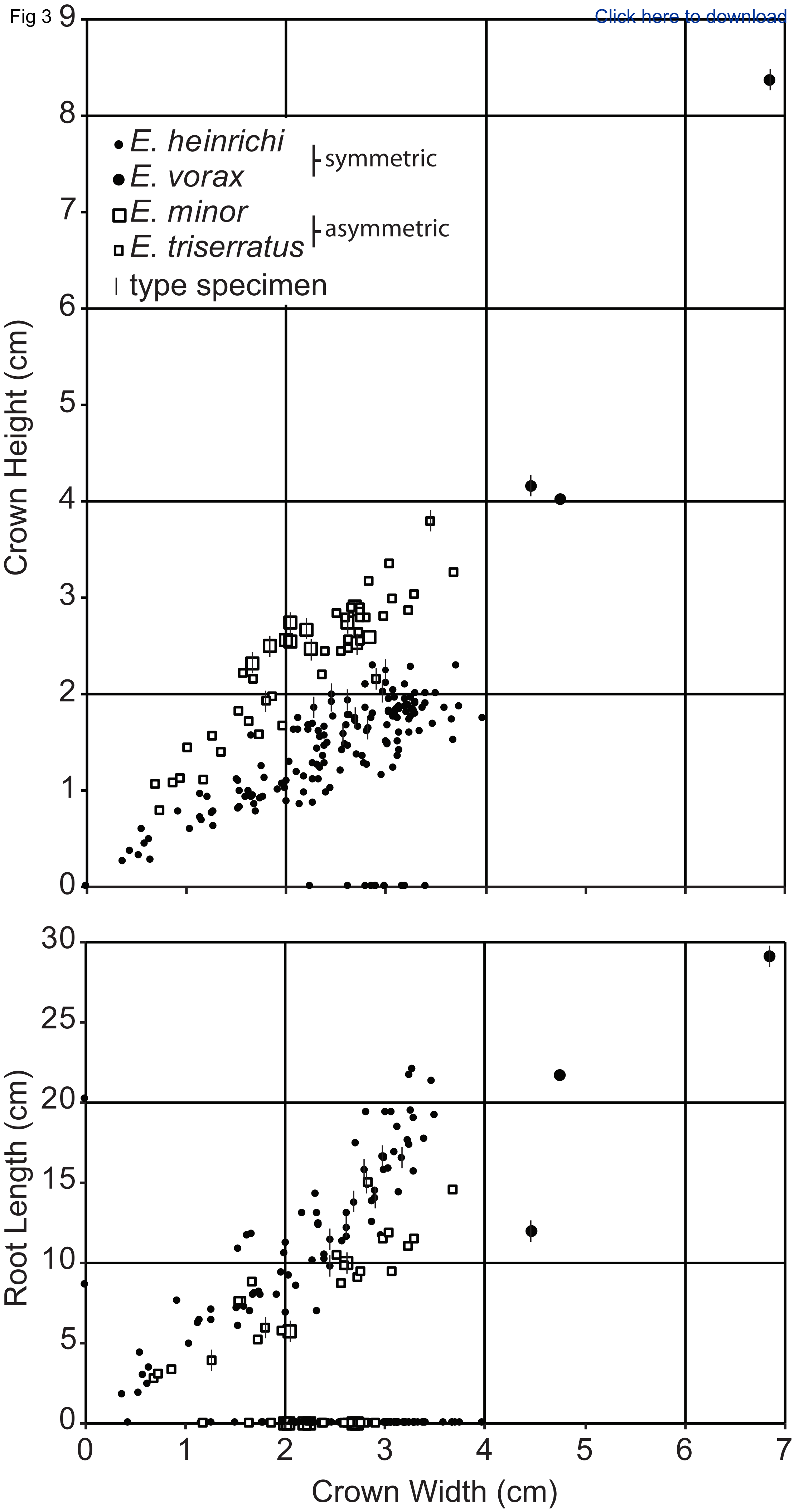

Fig 4

Click here to download Figure FIG4 EachSepSppPC1-PC2.eps

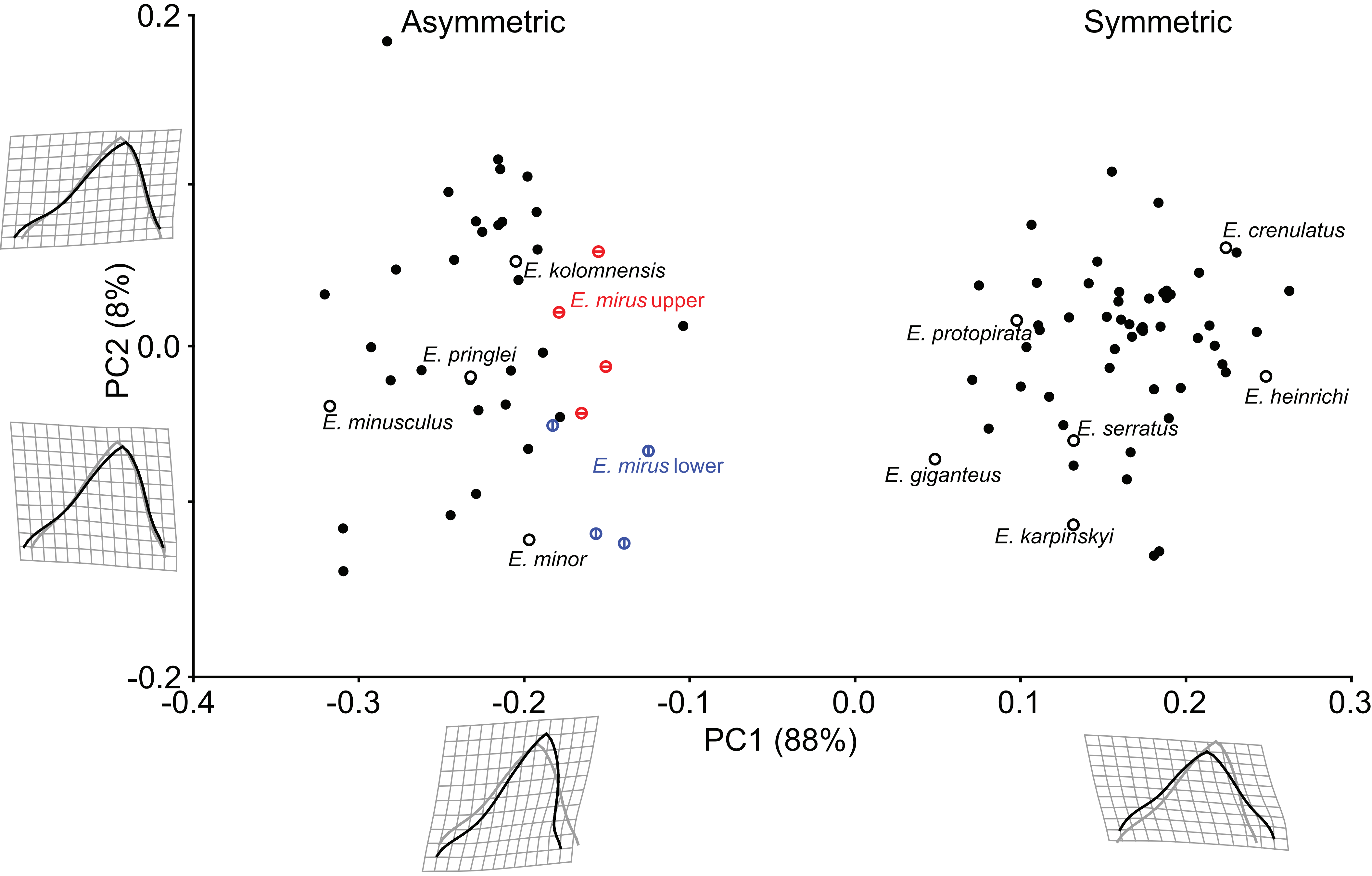

Fig 5

Click here to download Figure FIG5 SYM.eps

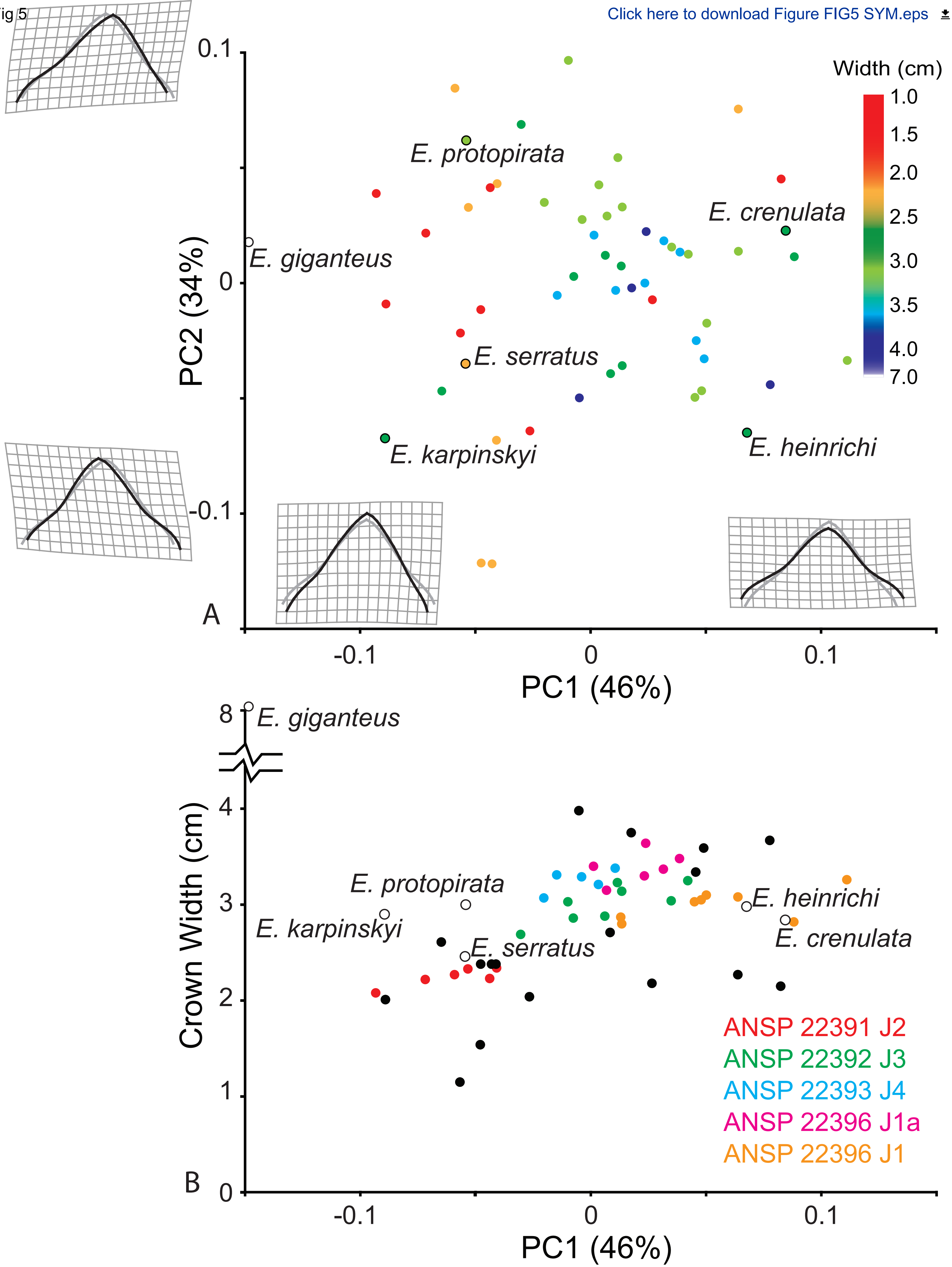

Fig 6

Click here to download Figure FIG6 ASYM.eps

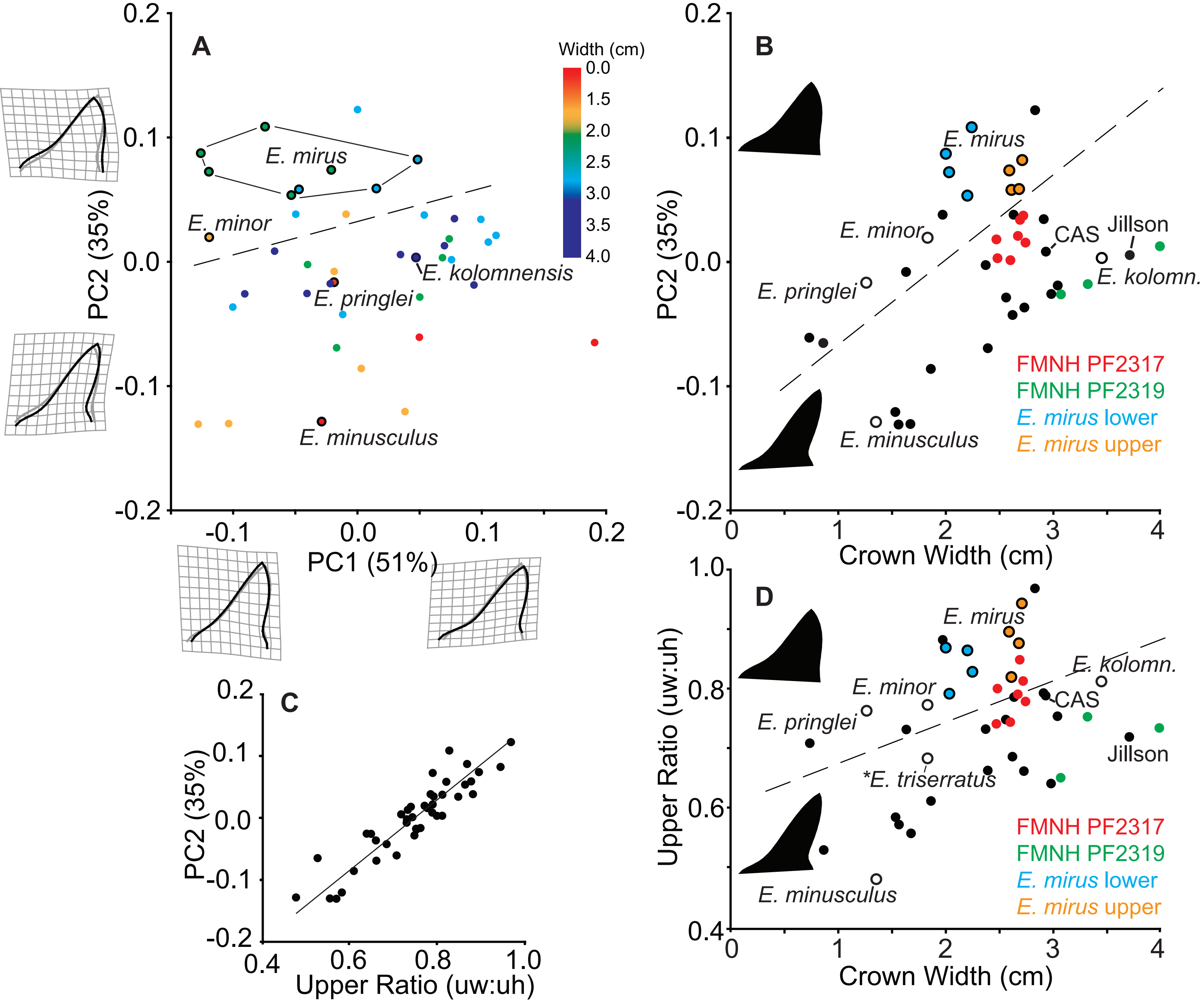

Fig 7

Click here to download Figure FIG7 Ed plate final.eps

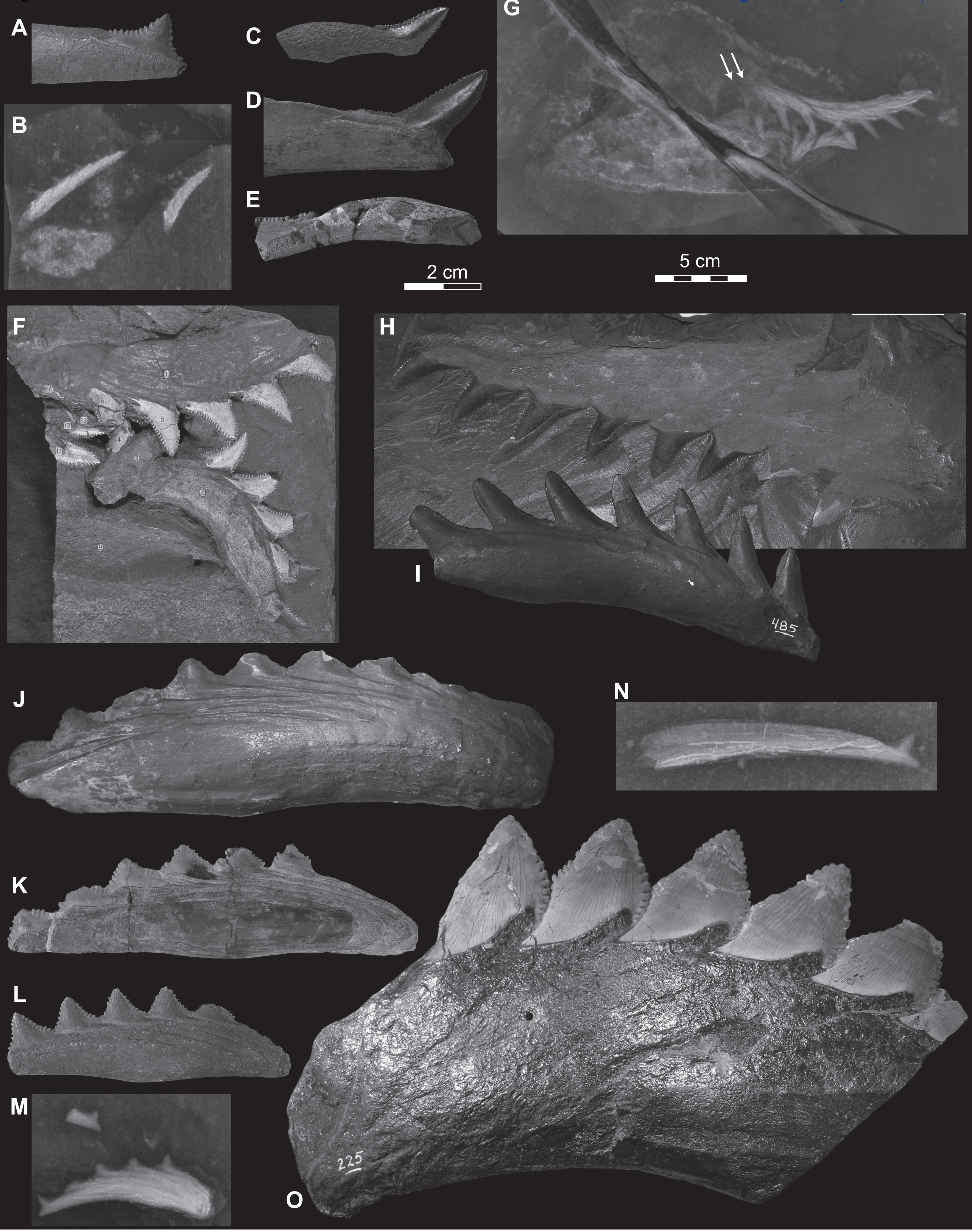

Click here to download Figure FIG8 Archetypes.eps

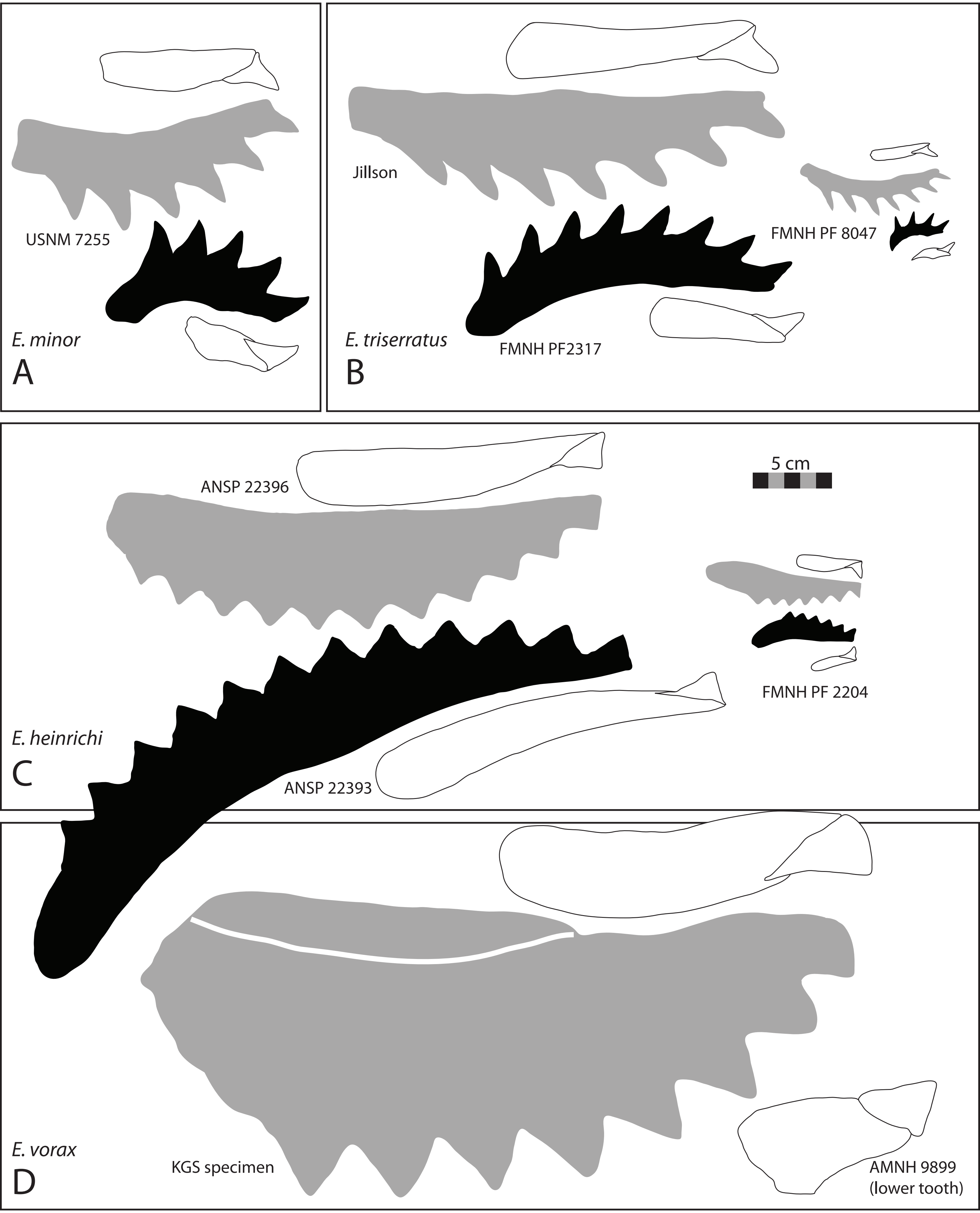

Fig 9

Click here to download Figure FIG9 SpaceTimeEd.eps

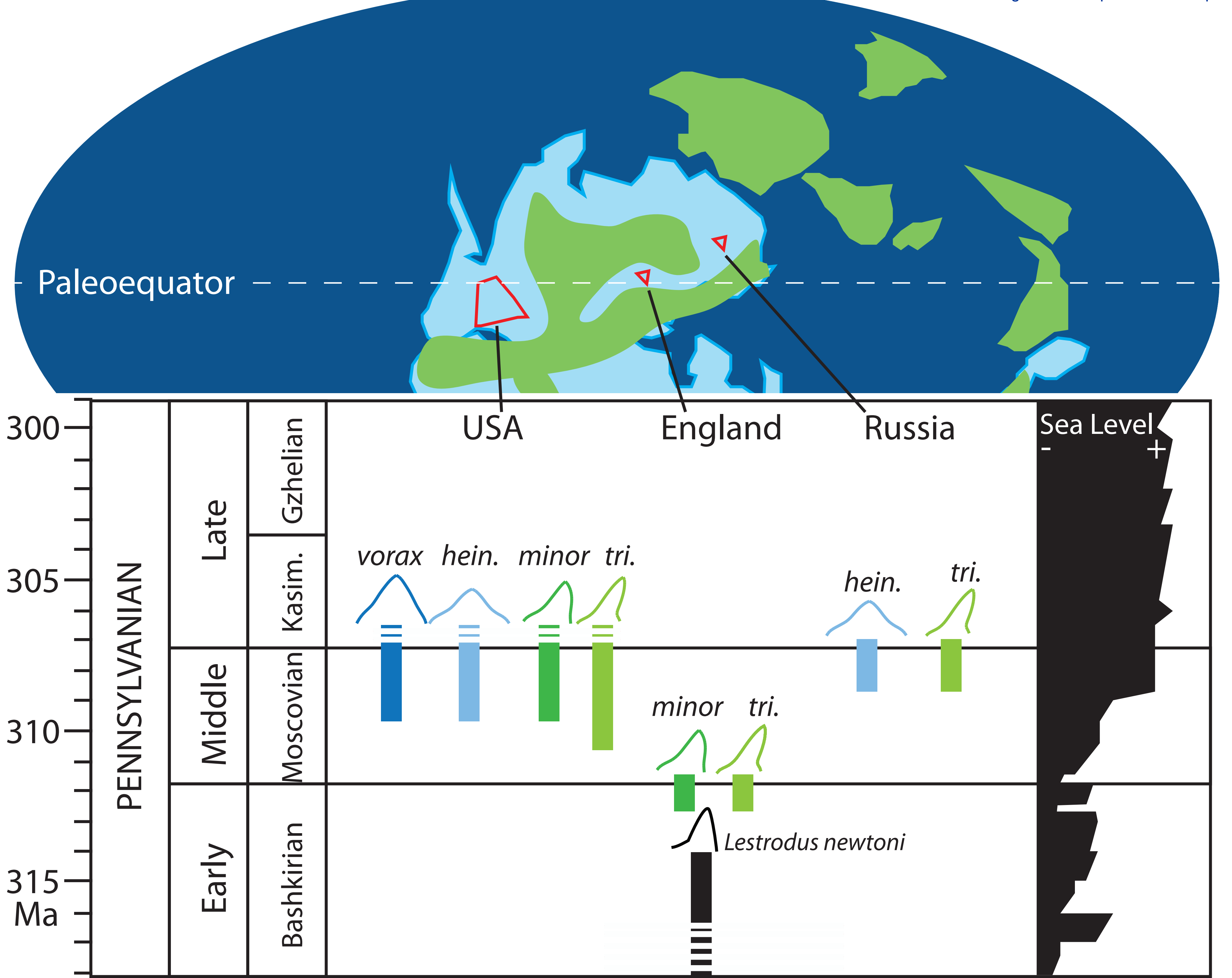

S1 Figure

Click here to access/download
Supporting Information
S1 Fig Jillson and ISM.eps

S2 Appendix Asymmetric XLS

Click here to access/download
Supporting Information
S2 Appendix ASYM.xlsx

S3 Appendix Symmetric XLS

Click here to access/download
Supporting Information
S3 Appendix SYM.xlsx
