# Supplementary material for: Redefining species concepts for the Pennsylvanian scissor tooth shark, Edestus
Source: PLoS One. 2019 Sep 4;14(9):e0220958. doi: 10.1371/journal.pone.0220958 (PMC6726245; doi:10.1371/journal.pone.0220958)
Supplement: Attachment — Submitted filename: Response to Reviewers - TapanilaPruitt.docx [file pone.0220958.s005.docx]

Response to Reviewers

Re: PONE-D-19-17544
Redefining species concepts for the Pennsylvanian scissor tooth shark, Edestus
PLOS ONE

Dear Academic Editor, Dr Carnevale,

We have considered the reviews and made substantial changes to the manuscript which addresses the suggestions, and we think has substantially improved the arguments and conclusions of the article.

Major modifications to the article include:

- Addition of statistical analyses to distinguish morphospaces (PERMANOVA, ANOSIM), to identify correlations (OLS) and to classify E. triserratus among asymmetric groupings (Discriminant analysis)
- Two new figures showing reconstructed Edestus and additional fossil whorls
- Improved the text throughout to resolve suggestions and critiques from the reviewers. These changes are listed below (blue)

Reviewer #1: I think that this is a very useful and clearly laid out paper that will be of considerable use. I have only a few quite minor comments-
L44. I suggest mention their affinities to the Holocephali (as opposed to sharks ss)

We added reference to Euchondrocephali. These comprise extinct members of the group we discuss in the article.

L48. I suggest the phrase “symphyseal tooth whorls” is used throughout, even at risk of repartition

Added “(whorls, hereafter). We think it would be too cumbersome to repeat the full phrase each time.

L60. I am not sure if the term “Shingles” is used much outside the US- certainly I have very rarely heard it. Would “roof tiles” be better?

Changed to “roof tiles”

L73. And post Palaeozoic too…

Removed “Paleozoic”

L85. It may be worth mentioning that a very similar situation exists with extinct species of myliobatid rays.

We did not include this as it seems tangential to the topic of the article.

L114. You have not stated the general stratigraphic position and many (including me) are not familiar with these stages. Also use both European (Late Carboniferous) and US (Pennysylvanian) general terms.

Added (Late Carboniferous, Pennsylvanian) here and at line 129

L136 x-rays

Corrected here and throughout text

L179 Do you mean youngest teeth? Is that because the roots are all growing on a single front?

We clarified this statement.

L223 May be better as an appendix as it disrupts the flow of the text

We did not make this change. We think the table will flow ok given PLOS ONEs formatting. If editor suggests so, we will move it to appendix.

L580 ?insert references

Added reference

L586 onwards. There should be some discussion of Itano’s microwear work. I know that at least one of the authors disagrees strongly with this, but it cannot be ignored and this may be an opportunity to discuss it.

We’ve added additional references to Itano’s work in the Discussion and argue that his findings confirm the anatomical reconstruction of slicing dentition.

Fig 7 seems rather dark and could be clearer. The mix of light and x-ray images is a bit confusing.

We’ve lightened the images overall to help this issue. We have also added a Figure of additional whorl specimens referred to in the text.

Reviewer #2: Dear authors,
I have included some comments and corrections directly into the attached PDF. Please consider these.

We made the syntax and grammatical corrections listed in the comments

Edestus heinrichi. A cast can’t be selected as holotype if the original holotype is lost! This certainly is not a proper procedure. The holotype has to be considered lost and a lectotype, e.g., needs to be established (if there is a syntype series). Please refer to the nomenclatural rules for this and check the taxonomic status of this species! There is a lot of material available.

We’ve corrected this oversight. The illustration in the original description of E. heinrichi is instead referable as the holotype. We provide explanation of this in the Systematics section including referral to ICZN rules.

- I would strongly recommend presenting the morphospace occupation of the various species as convex hulls. This would make it easier for the reader to see also possible overlaps in morphospace occupation.

We have done so for Figure 6, with explanation in the text as to how we defined the ‘groups’.

- You need to provide some statistic measures to support your interpretations from the geometric morphometric analyses such as multivariate analysis of variance (PERMANOVA) to test similarities in-group centroid position between different groups and the analysis of similarities (ANOSIM) to test quantitatively the degree of overlap between different groups.

We have presented both tests in the Summary of taxobases section. We lack enough specimens to do this evaluation for E. vorax, so instead rely on linear metrics to define it.

Reviewer #3: Dear Editor, Dear Authors,

1. The analysis based on linear measurements (paragraph “Linear measures of teeth”) has been performed in order to identify the mean variables and regression parameters useful to distinguish the four Edestus morphotypes/species. However, at this point it seems that authors have already identified the four taxobases a priori. In my opinion, this approach based on linear measurement should be performed AFTER the redefinition of the species concepts through geometric morphometrics, when the authors have already identified the four taxobases. In Figure 3 it is not clear if the other synonyms (E. protopirata, E. serratus, etc) plot close to the asymmetric or symmetric group.

We are clearer in our methods as to how we evaluated the species using first a split between asymmetric and symmetric forms using linear measures, then evaluating crown shape to discriminate our final species concepts. We’ve kept the order of presenting the data the same, i.e. linear measures before geometric.

2. I do not agree with the treatment of the data represented in Figure 3 and discussed in the paragraph “Linear measures of teeth”. The authors have included in the analysis some measurements that should have been treated as “missing”, that means putting “?” instead of “0.00” (see Table 2). In fact, all these specimens plot in the graphs (Fig 3) as all lying in the x-axes. This has inevitably effect on the regression analysis, leading to get different coefficients (slope and intercept), weaker correlation (low coefficient of determination r2) and, maybe, non-significant relationships between variables (btw: p-values are never shown or discussed in the text!). Moreover, since “zero” values are also present in some H and W values, I assume that they were also used to determine the mean of the ratio H:W. This must be avoided.

This has now been corrected in Figure 3. The correlations were originally done excluding zero data, but this wasn’t evident in our earlier presentation. We’ve also added OLS tests for r^2^ values.

3. There is a problem with using least squares regression for analysing raw data since they probably violate at least one of the assumptions of least squares regression: the equal variance. This may be detected in examination of the residuals. The data must have relatively equal dispersion around the regression line. However, at larger sizes, the raw data appear dispersed much more widely around the line compared to small sizes, leading to a triangle-shaped dispersion pattern (see e.g. E. heinrichi in Fig. 3). The authors should employ a weighted least squares regression or log-transform the data before performing the ordinary least squares analysis. Moreover, the coefficients of the regressions (or the equations themselves) along with r2 and p-values for each species, should be reported somewhere in order to aid future researchers in discriminating new species from these four ones.

We ran OLS on log transformed values and present those in the Summary of taxobases.

4. The terminology is sometimes ambiguous. In ichthyology, meristic features are countable structures of fishes (e.g. fin rays and spines, gill rakers, vertebrae, scales, etc). However, the authors often refer to meristic analyses or meristic approaches (see e.g. line 134) to indicate approaches and analyses based on linear measurements (CW, CH, RL, etc). I would suggest to use “traditional morphometrics” (based on linear measurements) instead of “meristic”. The authors are actually using traditional morphometric and geometric morphometric approaches.

We have removed usage of meristic in lieu of “traditional” or “linear measurements”

5. In the geometric morphometric analysis the authors should explain better how they defined the taxobases, at least for the asymmetric group. Although two ontogenetic series seem well separated and defined in the asymmetric group in Fig. 6B, the authors should clarify which criteria they used to separate the two groups through a dashed line. In my opinion some of the specimens (e.g. FMNH PF2317 or the black point just above) might fit with both groups. Moreover, why did the authors use PC2 instead of PC1 (which explains much more variation)?

We removed the dashed line and instead use concave hulls to define the morphospace of the “upper” and “lower” groups in Figure 4B & 4D. We revised the text to be more explicit in our decision to use PC2 (it varies with size, thus we can identify distinct ontogentic series), to define the two asymmetric groupings (including PF2317), and provide a better justification for including E. triserratus holotype within the ‘lower’ grouping based on discriminant analysis.

6. A further proofreading of the manuscript is needed to avoid small errors and typos. E.g.:
- Commas should be put after authorship, before the year (see e.g. line 360 but there are more)

Corrected

- I do not know if “equant” can be used to describe the shape of triangles. Do the authors mean “equilateral triangle”?

Corrected

- Use “Geometric Morphometrics” instead of “Geometric Morphometry” throughout the manuscript.

Corrected

- Line 222: “tallest” instead of “taller”?

corrected

- Line 275: “Principal component analysis” instead of “Principle components analysis”

Corrected

- In Table 2, crown uw, uh and uw:uh values are indicated for E. minor and E. triserratus but not for E. vorax and E. heinrichi. Is there any reason for that?

 	These were not measured because crown symmetry, H:W and RootL variables were sufficient to discriminate E. vorax and E. heinrichi from other Edestus specimens.
