# Supplementary material for: Redefining species concepts for the Pennsylvanian scissor tooth shark, Edestus
Source: PLoS One. 2019 Sep 4;14(9):e0220958. doi: 10.1371/journal.pone.0220958 (PMC6726245; doi:10.1371/journal.pone.0220958)
Supplement: Peer review history

# Peer review: Decision Letter 0

**DOI:** 10.1371/journal.pone.0220958.r001

**Authors:** Giorgio Carnevale

11 Jul 2019

PONE-D-19-17544

Redefining species concepts for the Pennsylvanian scissor tooth shark, Edestus

PLOS ONE

Dear Dr. Tapanila,

Thank you for submitting your manuscript to PLOS ONE. After careful consideration, we feel that it has merit but does not fully meet PLOS ONE’s publication criteria as it currently stands. Therefore, we invite you to submit a revised version of the manuscript that addresses the points raised during the review process.

We would appreciate receiving your revised manuscript by Aug 25 2019 11:59PM. When you are ready to submit your revision, log on to https://www.editorialmanager.com/pone/ and select the 'Submissions Needing Revision' folder to locate your manuscript file.

If you would like to make changes to your financial disclosure, please include your updated statement in your cover letter.

To enhance the reproducibility of your results, we recommend that if applicable you deposit your laboratory protocols in protocols.io, where a protocol can be assigned its own identifier (DOI) such that it can be cited independently in the future. For instructions see: http://journals.plos.org/plosone/s/submission-guidelines#loc-laboratory-protocols

Please include the following items when submitting your revised manuscript:

- A rebuttal letter that responds to each point raised by the academic editor and reviewer(s). This letter should be uploaded as separate file and labeled 'Response to Reviewers'.
- A marked-up copy of your manuscript that highlights changes made to the original version. This file should be uploaded as separate file and labeled 'Revised Manuscript with Track Changes'.
- An unmarked version of your revised paper without tracked changes. This file should be uploaded as separate file and labeled 'Manuscript'.

Please note while forming your response, if your article is accepted, you may have the opportunity to make the peer review history publicly available. The record will include editor decision letters (with reviews) and your responses to reviewer comments. If eligible, we will contact you to opt in or out.

We look forward to receiving your revised manuscript.

Kind regards,

Giorgio Carnevale, Ph.D

Academic Editor

PLOS ONE

Journal Requirements:

1. When submitting your revision, we need you to address these additional requirements.

Please ensure that your manuscript meets PLOS ONE's style requirements, including those for file naming. The PLOS ONE style templates can be found at

http://www.journals.plos.org/plosone/s/file?id=wjVg/PLOSOne_formatting_sample_main_body.pdf and http://www.journals.plos.org/plosone/s/file?id=ba62/PLOSOne_formatting_sample_title_authors_affiliations.pdf

2. Thank you for stating the following in the Acknowledgments Section of your manuscript:

 This research was partly conducted during sabbatical release time funded by Idaho State University.

We note that you have provided funding information that is not currently declared in your Funding Statement. However, funding information should not appear in the Acknowledgments section or other areas of your manuscript. We will only publish funding information present in the Funding Statement section of the online submission form.

Please remove any funding-related text from the manuscript and let us know how you would like to update your Funding Statement. Currently, your Funding Statement reads as follows:

The author(s) received no specific funding for this work.

Additional Editor Comments:

Dear Dr. Tapanila,

Your manuscript "Redefining species concepts for the Pennsylvanian scissor tooth shark, Edestus" has been assessed by three reviewers. Based on these reports, and my own assessment as Editor, I am pleased to inform you that it is potentially acceptable for publication in PLOS ONE, once you have carried out some essential revisions suggested by our reviewers, especially those conerning the use of geometric morphometric analyses.

Their reports, together with any other comments, are below.

We look forward to receiving your revised manuscript soon.

Best wishes,

Giorgio Carnevale

[Note: HTML markup is below. Please do not edit.]

Reviewers' comments:

Reviewer's Responses to Questions

**Comments to the Author**

1. Is the manuscript technically sound, and do the data support the conclusions?

The manuscript must describe a technically sound piece of scientific research with data that supports the conclusions. Experiments must have been conducted rigorously, with appropriate controls, replication, and sample sizes. The conclusions must be drawn appropriately based on the data presented. 

Reviewer #1: Yes

Reviewer #2: Partly

Reviewer #3: Yes

**********

2. Has the statistical analysis been performed appropriately and rigorously? 

Reviewer #1: Yes

Reviewer #2: No

Reviewer #3: No

**********

3. Have the authors made all data underlying the findings in their manuscript fully available?

The PLOS Data policy requires authors to make all data underlying the findings described in their manuscript fully available without restriction, with rare exception (please refer to the Data Availability Statement in the manuscript PDF file). The data should be provided as part of the manuscript or its supporting information, or deposited to a public repository. For example, in addition to summary statistics, the data points behind means, medians and variance measures should be available. If there are restrictions on publicly sharing data—e.g. participant privacy or use of data from a third party—those must be specified.

Reviewer #1: Yes

Reviewer #2: Yes

Reviewer #3: Yes

**********

4. Is the manuscript presented in an intelligible fashion and written in standard English?

PLOS ONE does not copyedit accepted manuscripts, so the language in submitted articles must be clear, correct, and unambiguous. Any typographical or grammatical errors should be corrected at revision, so please note any specific errors here.

Reviewer #1: Yes

Reviewer #2: Yes

Reviewer #3: Yes

**********

5. Review Comments to the Author

Please use the space provided to explain your answers to the questions above. You may also include additional comments for the author, including concerns about dual publication, research ethics, or publication ethics. (Please upload your review as an attachment if it exceeds 20,000 characters)

Reviewer #1: I think that this is a very useful and clearly laid out paper that will be of considerable use. I have only a few quite minor comments-

L44. I suggest mention their affinities to the Holocephali (as opposed to sharks ss)

L48. I suggest the phrase “symphyseal tooth whorls” is used throughout, even at risk of repartition

L60. I am not sure if the term “Shingles” is used much outside the US- certainly I have very rarely heard it. Would “roof tiles” be better?

L73. And post Palaeozoic too…

L85. It may be worth mentioning that a very similar situation exists with extinct species of myliobatid rays.

L114. You have not stated the general stratigraphic position and many (including me) are not familiar with these stages. Also use both European (Late Carboniferous) and US (Pennysylvanian) general terms.

L136 x-rays

L179 Do you mean youngest teeth? Is that because the roots are all growing on a single front?

L223 May be better as an appendix as it disrupts the flow of the text

L580 ?insert references

L586 onwards. There should be some discussion of Itano’s microwear work. I know that at least one of the authors disagrees strongly with this, but it cannot be ignored and this may be an opportunity to discuss it.

Fig 7 seems rather dark and could be clearer. The mix of light and x-ray images is a bit confusing.

Reviewer #2: Dear authors,

I have included some comments and corrections directly into the attached PDF. Please consider these. Some additional points should be considered:

- Edestus heinrichi. A cast can’t be selected as holotype if the original holotype is lost! This certainly is not a proper procedure. The holotype has to be considered lost and a lectotype, e.g., needs to be established (if there is a syntype series). Please refer to the nomenclatural rules for this and check the taxonomic status of this species! There is a lot of material available.

- I would strongly recommend presenting the morphospace occupation of the various species as convex hulls. This would make it easier for the reader to see also possible overlaps in morphospace occupation.

- You need to provide some statistic measures to support your interpretations from the geometric morphometric analyses such as multivariate analysis of variance (PERMANOVA) to test similarities in-group centroid position between different groups and the analysis of similarities (ANOSIM) to test quantitatively the degree of overlap between different groups.

Kind regards,

Jürgen Kriwet

Reviewer #3: Dear Editor, Dear Authors,

The manuscript of Tapanila and Pruitt is an interesting contribution to paleoichthyology since it redefines the species concept for one of the most enigmatic Paleozoic shark genera, Edestus, through quantitative methods, using a mix of traditional and geometric morphometric approaches. The manuscript is well written, figures are clear and the discussion is well addressed. However, in my opinion, the authors should address some questions mostly regarding the analyses performed on linear measurements.

1. The analysis based on linear measurements (paragraph “Linear measures of teeth”) has been performed in order to identify the mean variables and regression parameters useful to distinguish the four Edestus morphotypes/species. However, at this point it seems that authors have already identified the four taxobases a priori. In my opinion, this approach based on linear measurement should be performed AFTER the redefinition of the species concepts through geometric morphometrics, when the authors have already identified the four taxobases. In Figure 3 it is not clear if the other synonyms (E. protopirata, E. serratus, etc) plot close to the asymmetric or symmetric group.

2. I do not agree with the treatment of the data represented in Figure 3 and discussed in the paragraph “Linear measures of teeth”. The authors have included in the analysis some measurements that should have been treated as “missing”, that means putting “?” instead of “0.00” (see Table 2). In fact, all these specimens plot in the graphs (Fig 3) as all lying in the x-axes. This has inevitably effect on the regression analysis, leading to get different coefficients (slope and intercept), weaker correlation (low coefficient of determination r2) and, maybe, non-significant relationships between variables (btw: p-values are never shown or discussed in the text!). Moreover, since “zero” values are also present in some H and W values, I assume that they were also used to determine the mean of the ratio H:W. This must be avoided.

3. There is a problem with using least squares regression for analysing raw data since they probably violate at least one of the assumptions of least squares regression: the equal variance. This may be detected in examination of the residuals. The data must have relatively equal dispersion around the regression line. However, at larger sizes, the raw data appear dispersed much more widely around the line compared to small sizes, leading to a triangle-shaped dispersion pattern (see e.g. E. heinrichi in Fig. 3). The authors should employ a weighted least squares regression or log-transform the data before performing the ordinary least squares analysis. Moreover, the coefficients of the regressions (or the equations themselves) along with r2 and p-values for each species, should be reported somewhere in order to aid future researchers in discriminating new species from these four ones.

4. The terminology is sometimes ambiguous. In ichthyology, meristic features are countable structures of fishes (e.g. fin rays and spines, gill rakers, vertebrae, scales, etc). However, the authors often refer to meristic analyses or meristic approaches (see e.g. line 134) to indicate approaches and analyses based on linear measurements (CW, CH, RL, etc). I would suggest to use “traditional morphometrics” (based on linear measurements) instead of “meristic”. The authors are actually using traditional morphometric and geometric morphometric approaches.

5. In the geometric morphometric analysis the authors should explain better how they defined the taxobases, at least for the asymmetric group. Although two ontogenetic series seem well separated and defined in the asymmetric group in Fig. 6B, the authors should clarify which criteria they used to separate the two groups through a dashed line. In my opinion some of the specimens (e.g. FMNH PF2317 or the black point just above) might fit with both groups. Moreover, why did the authors use PC2 instead of PC1 (which explains much more variation)?

6. A further proofreading of the manuscript is needed to avoid small errors and typos. E.g.:

- Commas should be put after authorship, before the year (see e.g. line 360 but there are more)

- I do not know if “equant” can be used to describe the shape of triangles. Do the authors mean “equilateral triangle”?

- Use “Geometric Morphometrics” instead of “Geometric Morphometry” throughout the manuscript.

- Line 222: “tallest” instead of “taller”?

- Line 275: “Principal component analysis” instead of “Principle components analysis”

- In Table 2, crown uw, uh and uw:uh values are indicated for E. minor and E. triserratus but not for E. vorax and E. heinrichi. Is there any reason for that?

**********

6. PLOS authors have the option to publish the peer review history of their article (what does this mean?). If published, this will include your full peer review and any attached files.

If you choose “no”, your identity will remain anonymous but your review may still be made public.

**Do you want your identity to be public for this peer review?** For information about this choice, including consent withdrawal, please see our Privacy Policy.

Reviewer #1: Yes: Charlie Underwood

Reviewer #2: Yes: Juergen Kriwet

Reviewer #3: No

[NOTE: If reviewer comments were submitted as an attachment file, they will be attached to this email and accessible via the submission site. Please log into your account, locate the manuscript record, and check for the action link "View Attachments". If this link does not appear, there are no attachment files to be viewed.]

While revising your submission, please upload your figure files to the Preflight Analysis and Conversion Engine (PACE) digital diagnostic tool, https://pacev2.apexcovantage.com/. PACE helps ensure that figures meet PLOS requirements. To use PACE, you must first register as a user. Registration is free. Then, login and navigate to the UPLOAD tab, where you will find detailed instructions on how to use the tool. If you encounter any issues or have any questions when using PACE, please email us at figures@plos.org. Please note that Supporting Information files do not need this step.

Attachment Submitted filename: PONE-D-19-17544_JK.pdf Click here for additional data file.

# Author comment: Author response to Decision Letter 0

**DOI:** 10.1371/journal.pone.0220958.r002

25 Jul 2019

We have considered the reviews and made substantial changes to the manuscript which addresses the suggestions, and we think has substantially improved the arguments and conclusions of the article. 

Major modifications to the article include:

- Addition of statistical analyses to distinguish morphospaces (PERMANOVA, ANOSIM), to identify correlations (OLS) and to classify E. triserratus among asymmetric groupings (Discriminant analysis)

- Two new figures showing reconstructed Edestus and additional fossil whorls

- Improved the text throughout to resolve suggestions and critiques from the reviewers. These changes are listed below (blue)

Reviewer #1: I think that this is a very useful and clearly laid out paper that will be of considerable use. I have only a few quite minor comments-

L44. I suggest mention their affinities to the Holocephali (as opposed to sharks ss)

We added reference to Euchondrocephali. These comprise extinct members of the group we discuss in the article.

L48. I suggest the phrase “symphyseal tooth whorls” is used throughout, even at risk of repartition

Added “(whorls, hereafter). We think it would be too cumbersome to repeat the full phrase each time.

L60. I am not sure if the term “Shingles” is used much outside the US- certainly I have very rarely heard it. Would “roof tiles” be better?

Changed to “roof tiles”

L73. And post Palaeozoic too…

Removed “Paleozoic”

L85. It may be worth mentioning that a very similar situation exists with extinct species of myliobatid rays.

We did not include this as it seems tangential to the topic of the article.

L114. You have not stated the general stratigraphic position and many (including me) are not familiar with these stages. Also use both European (Late Carboniferous) and US (Pennysylvanian) general terms.

Added (Late Carboniferous, Pennsylvanian) here and at line 129

L136 x-rays

Corrected here and throughout text

L179 Do you mean youngest teeth? Is that because the roots are all growing on a single front?

We clarified this statement.

L223 May be better as an appendix as it disrupts the flow of the text

We did not make this change. We think the table will flow ok given PLOS ONEs formatting. If editor suggests so, we will move it to appendix.

L580 ?insert references

Added reference

L586 onwards. There should be some discussion of Itano’s microwear work. I know that at least one of the authors disagrees strongly with this, but it cannot be ignored and this may be an opportunity to discuss it.

We’ve added additional references to Itano’s work in the Discussion and argue that his findings confirm the anatomical reconstruction of slicing dentition. 

Fig 7 seems rather dark and could be clearer. The mix of light and x-ray images is a bit confusing.

We’ve lightened the images overall to help this issue. We have also added a Figure of additional whorl specimens referred to in the text.

Reviewer #2: Dear authors,

I have included some comments and corrections directly into the attached PDF. Please consider these. 

We made the syntax and grammatical corrections listed in the comments

Edestus heinrichi. A cast can’t be selected as holotype if the original holotype is lost! This certainly is not a proper procedure. The holotype has to be considered lost and a lectotype, e.g., needs to be established (if there is a syntype series). Please refer to the nomenclatural rules for this and check the taxonomic status of this species! There is a lot of material available.

We’ve corrected this oversight. The illustration in the original description of E. heinrichi is instead referable as the holotype. We provide explanation of this in the Systematics section including referral to ICZN rules.

- I would strongly recommend presenting the morphospace occupation of the various species as convex hulls. This would make it easier for the reader to see also possible overlaps in morphospace occupation.

We have done so for Figure 6, with explanation in the text as to how we defined the ‘groups’.

- You need to provide some statistic measures to support your interpretations from the geometric morphometric analyses such as multivariate analysis of variance (PERMANOVA) to test similarities in-group centroid position between different groups and the analysis of similarities (ANOSIM) to test quantitatively the degree of overlap between different groups.

We have presented both tests in the Summary of taxobases section. We lack enough specimens to do this evaluation for E. vorax, so instead rely on linear metrics to define it.

Reviewer #3: Dear Editor, Dear Authors,

1. The analysis based on linear measurements (paragraph “Linear measures of teeth”) has been performed in order to identify the mean variables and regression parameters useful to distinguish the four Edestus morphotypes/species. However, at this point it seems that authors have already identified the four taxobases a priori. In my opinion, this approach based on linear measurement should be performed AFTER the redefinition of the species concepts through geometric morphometrics, when the authors have already identified the four taxobases. In Figure 3 it is not clear if the other synonyms (E. protopirata, E. serratus, etc) plot close to the asymmetric or symmetric group. 

We are clearer in our methods as to how we evaluated the species using first a split between asymmetric and symmetric forms using linear measures, then evaluating crown shape to discriminate our final species concepts. We’ve kept the order of presenting the data the same, i.e. linear measures before geometric. 

2. I do not agree with the treatment of the data represented in Figure 3 and discussed in the paragraph “Linear measures of teeth”. The authors have included in the analysis some measurements that should have been treated as “missing”, that means putting “?” instead of “0.00” (see Table 2). In fact, all these specimens plot in the graphs (Fig 3) as all lying in the x-axes. This has inevitably effect on the regression analysis, leading to get different coefficients (slope and intercept), weaker correlation (low coefficient of determination r2) and, maybe, non-significant relationships between variables (btw: p-values are never shown or discussed in the text!). Moreover, since “zero” values are also present in some H and W values, I assume that they were also used to determine the mean of the ratio H:W. This must be avoided. 

This has now been corrected in Figure 3. The correlations were originally done excluding zero data, but this wasn’t evident in our earlier presentation. We’ve also added OLS tests for r2 values.

3. There is a problem with using least squares regression for analysing raw data since they probably violate at least one of the assumptions of least squares regression: the equal variance. This may be detected in examination of the residuals. The data must have relatively equal dispersion around the regression line. However, at larger sizes, the raw data appear dispersed much more widely around the line compared to small sizes, leading to a triangle-shaped dispersion pattern (see e.g. E. heinrichi in Fig. 3). The authors should employ a weighted least squares regression or log-transform the data before performing the ordinary least squares analysis. Moreover, the coefficients of the regressions (or the equations themselves) along with r2 and p-values for each species, should be reported somewhere in order to aid future researchers in discriminating new species from these four ones. 

We ran OLS on log transformed values and present those in the Summary of taxobases.

4. The terminology is sometimes ambiguous. In ichthyology, meristic features are countable structures of fishes (e.g. fin rays and spines, gill rakers, vertebrae, scales, etc). However, the authors often refer to meristic analyses or meristic approaches (see e.g. line 134) to indicate approaches and analyses based on linear measurements (CW, CH, RL, etc). I would suggest to use “traditional morphometrics” (based on linear measurements) instead of “meristic”. The authors are actually using traditional morphometric and geometric morphometric approaches.

We have removed usage of meristic in lieu of “traditional” or “linear measurements”

5. In the geometric morphometric analysis the authors should explain better how they defined the taxobases, at least for the asymmetric group. Although two ontogenetic series seem well separated and defined in the asymmetric group in Fig. 6B, the authors should clarify which criteria they used to separate the two groups through a dashed line. In my opinion some of the specimens (e.g. FMNH PF2317 or the black point just above) might fit with both groups. Moreover, why did the authors use PC2 instead of PC1 (which explains much more variation)?

We removed the dashed line and instead use concave hulls to define the morphospace of the “upper” and “lower” groups in Figure 4B & 4D. We revised the text to be more explicit in our decision to use PC2 (it varies with size, thus we can identify distinct ontogentic series), to define the two asymmetric groupings (including PF2317), and provide a better justification for including E. triserratus holotype within the ‘lower’ grouping based on discriminant analysis. 

6. A further proofreading of the manuscript is needed to avoid small errors and typos. E.g.:

- Commas should be put after authorship, before the year (see e.g. line 360 but there are more)

Corrected

- I do not know if “equant” can be used to describe the shape of triangles. Do the authors mean “equilateral triangle”?

Corrected

- Use “Geometric Morphometrics” instead of “Geometric Morphometry” throughout the manuscript.

Corrected

- Line 222: “tallest” instead of “taller”?

corrected

- Line 275: “Principal component analysis” instead of “Principle components analysis”

Corrected 

- In Table 2, crown uw, uh and uw:uh values are indicated for E. minor and E. triserratus but not for E. vorax and E. heinrichi. Is there any reason for that?

 These were not measured because crown symmetry, H:W and RootL variables were sufficient to discriminate E. vorax and E. heinrichi from other Edestus specimens.

Attachment Submitted filename: Response to Reviewers - TapanilaPruitt.docx Click here for additional data file.

# Editor report: Decision Letter 1

**DOI:** 10.1371/journal.pone.0220958.r003

**Authors:** Giorgio Carnevale

29 Jul 2019

Redefining species concepts for the Pennsylvanian scissor tooth shark, Edestus

PONE-D-19-17544R1

Dear Dr. Tapanila,

We are pleased to inform you that your manuscript has been judged scientifically suitable for publication and will be formally accepted for publication once it complies with all outstanding technical requirements.

Within one week, you will receive an e-mail containing information on the amendments required prior to publication. When all required modifications have been addressed, you will receive a formal acceptance letter and your manuscript will proceed to our production department and be scheduled for publication.

Shortly after the formal acceptance letter is sent, an invoice for payment will follow. To ensure an efficient production and billing process, please log into Editorial Manager at https://www.editorialmanager.com/pone/, click the "Update My Information" link at the top of the page, and update your user information. If you have any billing related questions, please contact our Author Billing department directly at authorbilling@plos.org.

If your institution or institutions have a press office, please notify them about your upcoming paper to enable them to help maximize its impact. If they will be preparing press materials for this manuscript, you must inform our press team as soon as possible and no later than 48 hours after receiving the formal acceptance. Your manuscript will remain under strict press embargo until 2 pm Eastern Time on the date of publication. For more information, please contact onepress@plos.org.

With kind regards,

Giorgio Carnevale, Ph.D

Academic Editor

PLOS ONE

# Editor report: Acceptance letter

**DOI:** 10.1371/journal.pone.0220958.r004

**Authors:** Giorgio Carnevale

23 Aug 2019

PONE-D-19-17544R1 

Redefining species concepts for the Pennsylvanian scissor tooth shark, *Edestus*

Dear Dr. Tapanila:

I am pleased to inform you that your manuscript has been deemed suitable for publication in PLOS ONE. Congratulations! Your manuscript is now with our production department. 

If your institution or institutions have a press office, please notify them about your upcoming paper at this point, to enable them to help maximize its impact. If they will be preparing press materials for this manuscript, please inform our press team within the next 48 hours. Your manuscript will remain under strict press embargo until 2 pm Eastern Time on the date of publication. For more information please contact onepress@plos.org.

For any other questions or concerns, please email plosone@plos.org. 

Thank you for submitting your work to PLOS ONE.

With kind regards,

PLOS ONE Editorial Office Staff

on behalf of

Dr. Giorgio Carnevale 

Academic Editor

PLOS ONE
